# A Small Molecule Agonist of EphA2 Receptor Tyrosine Kinase Inhibits Tumor Cell Migration In Vitro and Prostate Cancer Metastasis In Vivo

**DOI:** 10.1371/journal.pone.0042120

**Published:** 2012-08-15

**Authors:** Aaron Petty, Eugene Myshkin, Haina Qin, Hong Guo, Hui Miao, Gregory P. Tochtrop, Jer-Tsong Hsieh, Phillip Page, Lili Liu, Daniel J. Lindner, Chayan Acharya, Alexander D. MacKerell, Eckhard Ficker, Jianxing Song, Bingcheng Wang

**Affiliations:** 1 Rammelkamp Center for Research and Department of Medicine, MetroHealth Campus, Case Western Reserve University School of Medicine, Cleveland, Ohio, United States of America; 2 Departments of Biological Sciences, Faculty of Science, National University of Singapore, Singapore, Singapore; 3 Department of Chemistry, Case Western Reserve University, Cleveland, Ohio, United States of America; 4 Case Comprehensive Cancer Center, Case Western Reserve University School of Medicine, Cleveland, Ohio, United States of America; 5 Department of Urology, University of Texas Southwestern Medical Center, Dallas, Texas, United States of America; 6 Reichert, Inc., Depew, New York, United States of America; 7 Department of Medicine, Division of Hematology and Oncology, University Hospitals Case Medical Center, Case Western Reserve University School of Medicine, Cleveland, Ohio, United States of America; 8 Taussig Cancer Institute, Cleveland Clinic Foundation, Cleveland, Ohio, United States of America; 9 Department of Pharmaceutical Sciences, School of Pharmacy, University of Maryland, Baltimore, Maryland, United States of America; 10 Department of Biochemistry, Yong Loo Lin School of Medicine, National University of Singapore, Singapore, Singapore; 11 Department of Pharmacology, Case Western Reserve University School of Medicine, Cleveland, Ohio, United States of America; Queensland University of Technology, Australia

## Abstract

During tumor progression, EphA2 receptor can gain ligand-independent pro-oncogenic functions due to Akt activation and reduced ephrin-A ligand engagement. The effects can be reversed by ligand stimulation, which triggers the intrinsic tumor suppressive signaling pathways of EphA2 including inhibition of PI3/Akt and Ras/ERK pathways. These observations argue for development of small molecule agonists for EphA2 as potential tumor intervention agents. Through virtual screening and cell-based assays, we report here the identification and characterization of doxazosin as a novel small molecule agonist for EphA2 and EphA4, but not for other Eph receptors tested. NMR studies revealed extensive contacts of doxazosin with EphA2/A4, recapitulating both hydrophobic and electrostatic interactions recently found in the EphA2/ephrin-A1 complex. Clinically used as an α1-adrenoreceptor antagonist (Cardura®) for treating hypertension and benign prostate hyperplasia, doxazosin activated EphA2 independent of α1-adrenoreceptor. Similar to ephrin-A1, doxazosin inhibited Akt and ERK kinase activities in an EphA2-dependent manner. Treatment with doxazosin triggered EphA2 receptor internalization, and suppressed haptotactic and chemotactic migration of prostate cancer, breast cancer, and glioma cells. Moreover, in an orthotopic xenograft model, doxazosin reduced distal metastasis of human prostate cancer cells and prolonged survival in recipient mice. To our knowledge, doxazosin is the first small molecule agonist of a receptor tyrosine kinase that is capable of inhibiting malignant behaviors *in vitro* and *in vivo*.

## Introduction

As a member of the erythropoietin-producing hepatocellular (Eph) subfamily of receptor tyrosine kinases (RTKs), EphA2 was originally called epithelial cell kinase, or Eck, due to its widespread expression in epithelial cells *in vitro* and *in vivo* [1]. Subsequent studies revealed that EphA2 was overexpressed in human cancers, and that overexpression was correlated with malignant progression and poor prognosis [2], [3]. A large number of studies have demonstrated that EphA2 overexpression and activation promote tumorigenesis, suggesting a potential role as an oncogene [4], [5], [6], [7], [8]. Overexpression of EphA2 in breast epithelial cells induced morphological transformation [8], while in prostate cancer and glioma cell lines, elevated EphA2 expression caused increased chemotactic cell migration and invasion [9].

Contrasting the pro-oncogenic roles, many studies have shown that EphA2 activation by its ligand, ephrin-A1, regulates cellular behaviors in a manner more consistent with it being a tumor suppressor, including induction of apoptosis, inhibition of cell proliferation, and suppression of cell migration [7], [10], [11], [12]. *In vivo* studies demonstrate that EphA2 activation by systemically administered ephrin-A1 decreases tumorigenicity and invasiveness of carcinoma xenografts [13], [14]. Moreover, EphA2 deletion mice show increased susceptibility to carcinogen-induced skin tumorigenesis [15].

Recent studies are beginning to shed light on the paradoxical observations [3], [16]. It is revealed that EphA2 receptor has diametrically opposite roles in tumorigenesis [9]. Upon ligand stimulation, EphA2 inhibits cell migration in keeping with the well-established repulsive roles of Eph receptors in regulating cell motility [17], [18]. In direct contrast, in the absence of ligand, EphA2 promotes cell migration, which is correlated with its expression level. Mechanistically, EphA2 is found to be a substrate of Akt that is activated in different human cancers [9], [19]. Akt phosphorylates EphA2 on serine 897 located in the well-exposed loop between kinase domain and sterile-α motif (SAM). Mutagenesis, pharmacological and cellular studies show S897 phosphorylation is essential for migration-stimulatory effects of the EphA2 in the absence of ligand [9]. EphA2 overexpression is often accompanied by loss of expression or mislocalization of ephrin-A1 in breast cancer [20], glioma [21] and skin tumors [15]. The reduced ephrin-A expression coupled with increased EphA2 expression and frequent Akt activation provide a permissive environment to promote ligand-independent pro-invasive Akt-EphA2 crosstalk, which may be in part responsible for EphA2 overexpression during tumor progression and the correlation of EphA2 expression and unfavorable prognosis. Supporting this notion, immunohistochemical examination of human glioma specimens with an antibody against the phospho-S897 revealed that activation of Akt-EphA2 signaling is associated with malignant progression [9].

**Figure 1:**
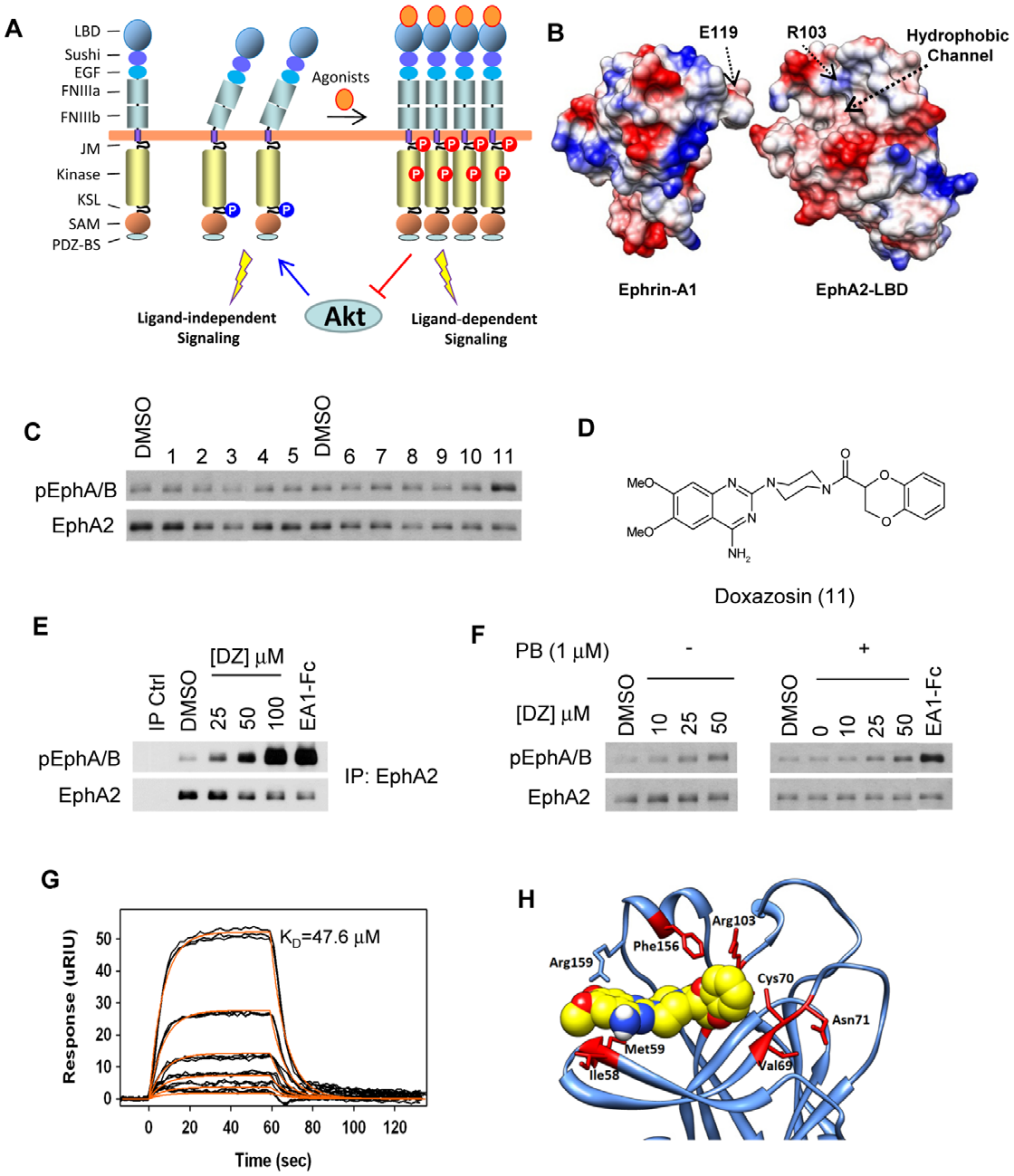
*In silico* screening identifies doxazosin as a novel agonist for EphA2 receptor. (A) Schematic illustration of the predicted effects of small molecule agonists in inducing ligand-dependent signaling. (B) Crystal structure of the EphA2 ligand binding domain (LBD) in complex with ephrin-A1. Highlighted are the hydrophobic pocket and arginine 103 of the EphA2-LBD that interact with the G–H loop of ephrin-A1 and glutamate 119 of ephrin-A1, respectively. EphA2-LBD was rotated ∼10° counter-clockwise to better reveal the binding pocket. (C) Small molecule screening identifies doxazosin (Compound 11) as a novel EphA2 agonist. MDA-231-A2 cells were treated with Compounds 1–11 (50 µM in 0.2% DMSO) for 30 minutes and cell lysates were subject to immunoblot for phosphorylated EphA/B kinases (pEphA/B) and total EphA2. (D) Chemical structure of doxazosin (DZ). (E) Dose-response of EphA2 activation by DZ. MDA-231-A2 cells were treated with the indicated doses of DZ for 30 minutes and lysates were immunoprecipitated with an EphA2-specific antibody and blotted as in (C). Treatment with 1 µg/ml ephrin-A1-Fc (EA1-Fc) for 10 minutes served as a positive control. Note decreasing amount of EphA2 following ephrin-A1 and doxazosin treatment. (F) Immunoblots for pEphA/B on lysates from MDA-231-A2 cells pretreated with 1 µM phenoxybenzamine and then treated for 1 hour with indicated doses of DZ. Treatment with 1 µg/ml ephrin-A1-Fc (EA1-Fc) served as a positive control. Treatment with 0.2% DMSO for either 1 hour (left), or 5 hours (right) served as vehicle controls. (G) Representative plot from Surface Plasmon Resonance (SPR) analysis of DZ binding to the recombinant ligand binding domain of EphA2. Curves from bottom to top represent concentrations of 1.56, 3.13, 6.25, 12.5, 25, 50 µM. Determined K_D_ value is shown within plot. (H) Molecular modeling of surface area diagram indicating amino acids of EphA2 potentially involved in direct interaction with doxazosin. The four amino acids of the ephrin-A1 loop are shown in red. Images were created using UCSF Chimera.

Importantly, ligand stimulation of tumor cells in vitro inactivates Akt and causes dephosphorylation of EphA2 on S897 [9], pointing to the intricate dichotomy of EphA2 functions, i.e., ligand-dependent tumor suppression and ligand-independent tumor promotion. Other tumor suppressor functions of EphA2 are also activated upon ligand-induced EphA2 activation, including inactivation of the Ras/ERK pathway. The ligand-dependent signaling culminates in the inhibition of cell migration and proliferation, although the specific responses are modulated by cellular context, such as Ras activation status in a given tumor cell type. These studies motivated us to propose that small molecule agonists for EphA2 can be exploited as novel cancer therapeutics. As illustrated in Figure 1A, such agonists may not only sever the pro-oncogenic Akt-EphA2 crosstalk, but also re-activate intrinsic ligand-dependent tumor suppressor functions of EphA2.

In this study, we have sought to identify small molecule agonists using a combination of structure-based virtual screening and cell-based assays. We report the discovery and characterization of doxazosin as a novel agonist for EphA2 and EphA4. Moreover, in a newly established orthotopic xenograft model of metastatic prostate cancer, systemic administration of doxazosin significantly suppressed distal metastasis and prolonged overall survival. To our knowledge, this is the first example of a small molecule RTK agonist with anti-cancer efficacy *in vitro* and *in vivo*.

## Results

### Structure-based virtual screening and cell-based assays identifies doxazosin as an agonist capable of inducing catalytic activation of EphA2 receptor tyrosine kinase

To identify small molecule agonists for EphA2, we took a structure-based *in silico* screening approach. Our molecular modeling of the EphA2 ligand-binding domain (LBD) based on the crystal structure of the EphB2 LBD [22] revealed that the binding pocket of EphA2 can incorporate up to 4 amino acids, suggesting that it could accommodate small molecules with a molecular weight (MW) of about 500 Dalton [23]. This notion was confirmed recently by determination of the crystal structure of the EphA2/ephrin-A1 complex [24] (Figure 1B). The size falls in the range of common drugs [25], making EphA2 LBD a desirable target for drug discovery. Toward this end, we initiated *in silico* screening to search for small molecules that interact favorably with the ligand-binding pocket of EphA2 derived from molecular modeling [23] before the crystal structure became available. Multiple conformations for each structure were generated with OMEGA (OpenEye) and each conformation was docked individually using DOCK [26]. Our initial screening of over 750,000 compounds identified a number of small molecules that could potentially interact with the ligand-binding pocket.

The commercially available top-scoring compounds were screened for their ability to induce EphA2 activation in MDA-MB-231 breast cancer cells. Because MDA-MB-231 cells express endogenous EphA2 as well as other Eph receptors, including EphA1, we overexpressed EphA2 (Figure S1) in order to minimize the contribution from other Eph receptors in our analysis. The cells were stimulated with the compounds at 50 µM. Total cell lysates were probed with a previously described antibody that recognizes the activated Eph receptors [27]. Figure 1C shows the results from a representative subset of compounds with structures given in Figure S2. Among the small molecules tested, compound 11, or doxazosin, activated EphA2 to the greatest extent. Originally developed as an antagonist for α1-adrenoreceptor, doxazosin (Figure 1D) is an FDA-approved drug (Cardura®) commonly used clinically for treating hypertension and benign prostate hyperplasia (BPH). To confirm specific EphA2 activation by doxazosin, we analyzed levels of activated Eph receptor in MDA-231-A2 cells following treatment with multiple doses of doxazosin and EphA2 immunoprecipitation. Doxazosin activated EphA2 receptor in a dose-dependent manner. Activation was detectable at 25 µM and became stronger at 50 µM or higher (Figure 1E). Similar to the native ligands, there was also degradation of EphA2 following exposure to high doses of doxazosin, which is characteristic of most RTKs upon ligand-induced activation including Ephs [42].

### Doxazosin induces catalytic activation of EphA2 independent of α1-adrenoreceptor antagonism

Because doxazosin is a well-characterized antagonist of α1-adrenoreceptor, a question arose whether EphA2 catalytic activation by doxazosin might be due to an indirect effect of its α1-adrenoreceptor antagonism. To address this question, we pretreated MDA-231-A2 cells with the well-characterized irreversible α1-adrenoreceptor inhibitor, phenoxybenzamine [28], [29], [30], and then assayed for induction of EphA2 phosphorylation by doxazosin. MDA-231-A2 cells were chosen because they have previously been shown to express both α1a and α1b–adrenoreceptors [31]. No difference in EphA2 activation by doxazosin was observed following phenoxybenzamine pretreatment versus no pretreatment (Figure 1F), demonstrating that doxazosin directly activates EphA2 independent of its α1-adrenoreceptor antagonism.

Two other top-scoring compounds, dobutamine and labetalol (Compounds 9 and 10), function as a β1-adrenoreceptor agonist and an α/β-adrenoreceptor antagonist, respectively. Treatment with dobutamine failed to activate EphA2 up to 500 µM, while labetalol failed to activate even at 500 µM, confirming that doxazosin is indeed more potent and that activation is not a direct result of general adrenoreceptor binding (Figure S3A).

### Doxazosin directly interacts with the EphA2 LBD

Because doxazosin was discovered via virtual screening targeting the ligand binding pocket of EphA2, it was expected to directly bind to the domain. To test this, binding of doxazosin to the previously described recombinant LBD of EphA2 [24] was analyzed using Surface Plasmon Resonance (SPR). We found that doxazosin directly bound to the EphA2 LBD with a dissociation constant (K_D_) of 47.6 µM (Figure 1G). Binding of both dobutamine and labetalol was also tested and shown to occur with much lower affinity than doxazosin (K_D_ = 1.5 mM and 0.44 mM, respectively) (Figure S3B). Taken together, these results demonstrate that doxazosin directly binds to the EphA2 LBD.

Upon the recent determination of the X-ray crystal structure of the EphA2-LBD in complex with ephrin-A1 [24], we compared it with the homology model of EphA2 [23] used in the original screening. Despite some expected differences, the EphA2 binding site of the homology model was overall similar to the one in the crystal structure (Figure S4), supporting general validity of the molecular model for the virtual screening. Next, we repeated the docking of doxazosin into the ligand binding site from the EphA2 crystal structure. Figure 1H shows doxazosin docked into the EphA2 crystal structure, in an orientation similar to that in the NMR structure of the EphA2-doxazosin complex (below). A new round of virtual screening was also conducted using the EphA2 LBD crystal structure. However, of the 30 new top-scoring compounds tested, we did not find agonists that displayed better activities than doxazosin (not shown).

### Doxazosin activates EphA2 receptor in different cell types

**Figure 2:**
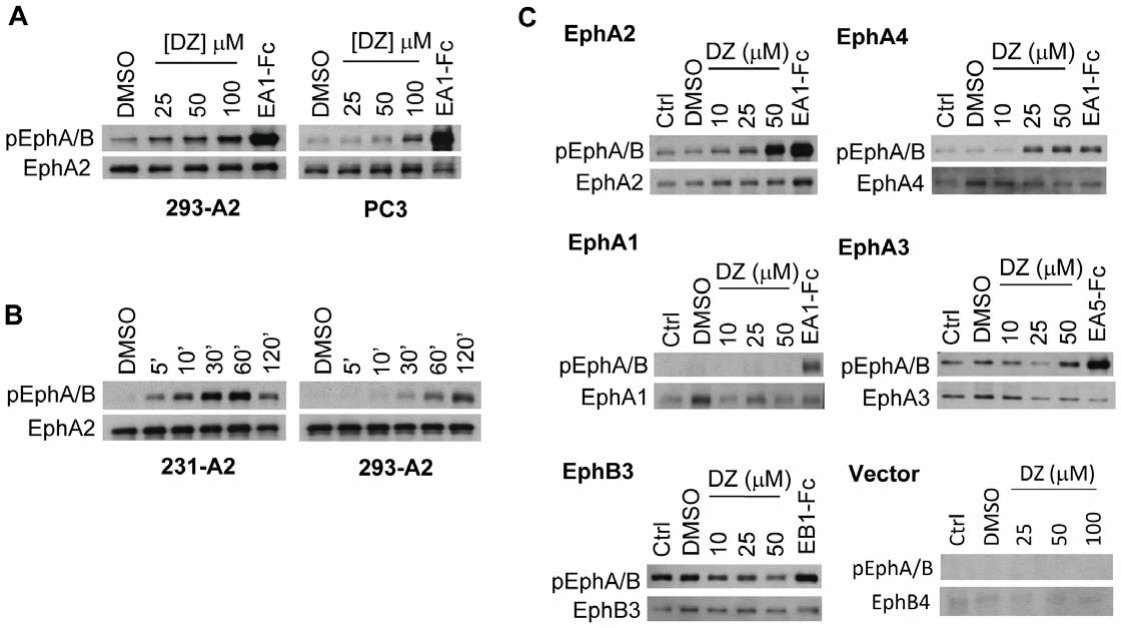
Doxazosin activates EphA2 receptor in different cell types. (A) Immunoblots for pEphA/B on lysates from PC-3 and HEK 293-A2 (293-A2) cell lines treated for 30 minutes with indicated doses of doxazosin (DZ). (B) Immunoblots for pEphA/B on lysates from MDA-231-A2 (231-A2) and 293-A2 cell lines treated with 50 µM DZ in 0.2% DMSO for indicated times. (C) Doxazosin selectively activates EphA2 and EphA4 receptors. Immunoblots for pEphA/B on lysates from HEK 293 cell lines expressing given Eph receptors following treatment with indicated doses of doxazosin (DZ) for 60 minutes. Treatment with 1 µg/ml ephrin-A1-Fc ligand (EA1-Fc) for 10 minutes (EphA2) and 30 minutes (Vector, EphA1, EphA4), as well as 30 minute treatment with ephrin-A5-Fc (EA5-Fc) (EphA3) and ephrin-B1-Fc (EB1-Fc) (EphB3) served as positive controls. Blotting for total Eph kinases served as loading controls.

To determine whether the EphA2 agonist activity of doxazosin is cell-context specific, we evaluated the effects of doxazosin on additional cell types. We first utilized HEK 293 cells overexpressing EphA2 (HEK 293-A2) that we described previously [9]. HEK 293 cells express low levels of endogenous EphA receptors [12]; overexpression of EphA2 in these cells allows further demonstration of specific activation of the exogenous kinase. Similar to MDA-231-A2 cells, significant activation of EphA2 was seen in HEK 293-A2 cells upon treatment with doxazosin starting at 25 µM (Figure 2A). Next we tested PC-3 cells that express high levels of endogenous EphA2 receptor [32]. Doxazosin also activated endogenous EphA2 in PC-3 cells, although it was not evident until 50 µM. The different kinetics among these cell types may be due, in part, to the different expression levels of EphA2 in the three different cell lines or the specific cellular context. In addition, EphA2 activation in PC-3 cells further supports the α1-adrenoreceptor-independent mechanism, as these cells lack detectable α1a-adrenoreceptor expression [33].

We next evaluated the time-course of activation in both MDA-231-A2 and HEK 293-A2 cells upon treatment with a single 50 µM dose of doxazosin. Significant EphA2 activation could be detected as early as 5 min after treatment in the MDA-231-A2 cell line, which peaked around 30 min (Figure 2B). EphA2 activation also became evident after 10 min treatment with doxazosin in HEK 293-A2 cells, although it followed a slower kinetics.

### Doxazosin preferentially activates EphA2 and EphA4 kinases

There are 14 mammalian Eph receptors that share significant sequence homology [34]. This led us to investigate whether doxazosin may also activate other Eph receptors. For this purpose, HEK 293 cell lines overexpressing EphA1, EphA2, EphA3, EphA4, and EphB3 kinases were utilized. We found that doxazosin activated both EphA2 and EphA4 kinases following a similar dose-response relationship (Figure 2C). However, no activation of EphA1 was seen at the same concentrations, and activation of EphA3 was weak compared to that of EphA2 and EphA4. EphB3 has higher basal levels of activation (Figure 2C), which was not further activated. In fact, there was a notable decrease in EphB3 activation upon doxazosin exposure. There is also a moderate level of endogenous EphB4 expression in HEK 293 cells; the lack of activated Eph kinase signals following doxazosin exposure in vector control cells indicated that the endogenous EphB4 was not activated either (Figure 2C). This data shows that doxazosin preferentially activates EphA2 and EphA4 among the various Eph receptors tested.

### NMR structure reveals extensive direct interactions of doxazosin with EphA4

The dual selectivity of doxazosin for EphA2 and EphA4 opened up a possibility to investigate the structural basis of the interactions using NMR spectroscopy. Because the EphA2 LBD expressed in *E. coli* was completely insoluble and could not be refolded after repeated attempts, we focused on the interactions of the EphA4 LBD with doxazosin. First, we assessed EphA4 binding to doxazosin by isothermal calorimetry. The dissociation constant (K_D_) was calculated to be 12.4 µM (Figure S5A), similar to that of EphA2/doxazosin interactions measured by SPR (Figure 1G). Far-UV circular dichroism showed that doxazosin binding induces no significant secondary structure change in the EphA4 LBD (Figure S5B).

**Figure 3:**
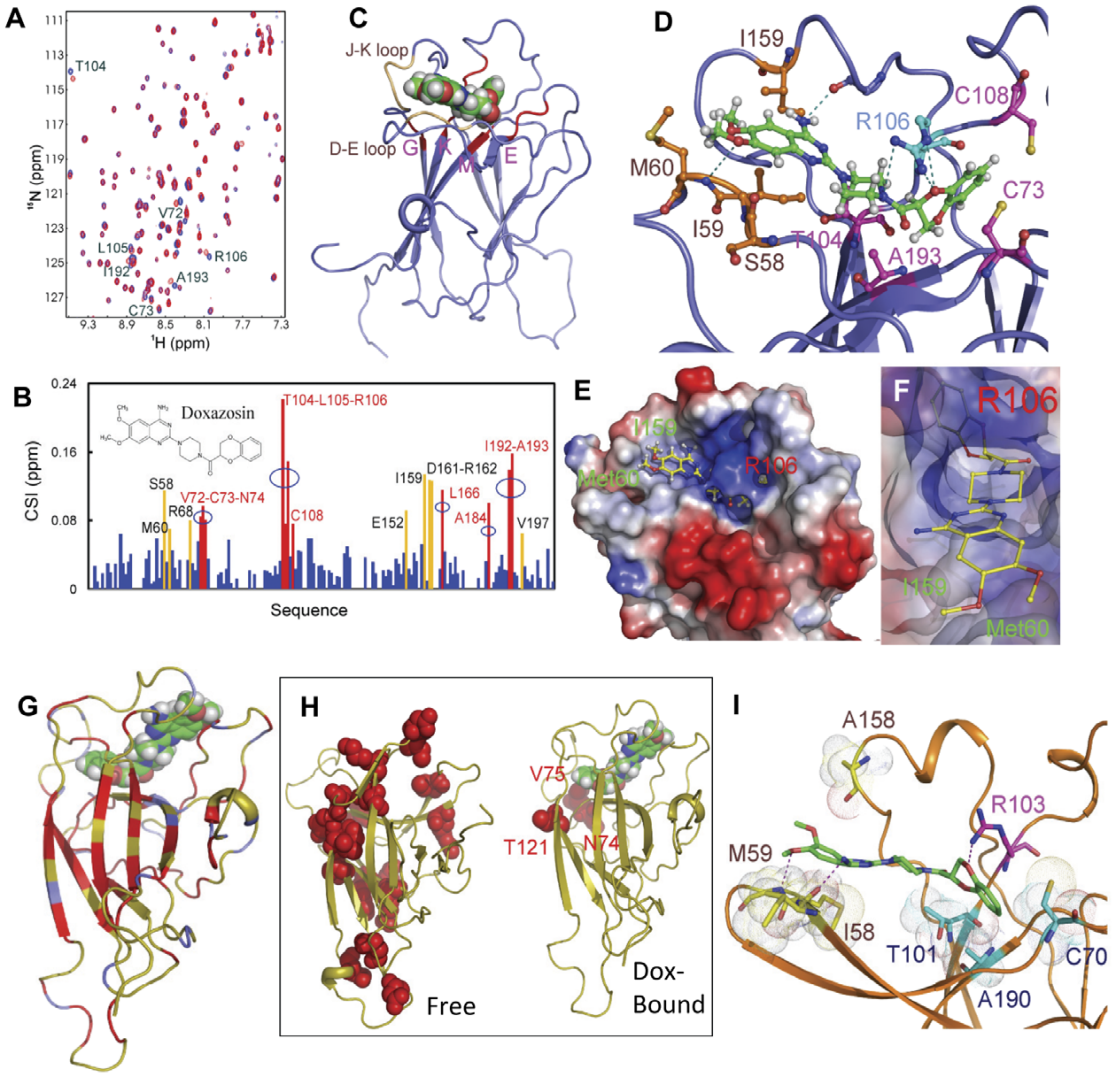
Structure and dynamics of the EphA4-doxazosin complex. (A) ^1^H-^15^N NMR HSQC spectra of the EphA4 LBD in the absence (blue) and in the presence (red) of doxazosin (DZ) at a molar ratio of 1∶5 (EphA4∶DZ). Several residues located over the convex surface of the EphA4 ephrin-binding channel are labeled. (B) Residue-specific chemical shift index (CSI) of the EphA4 LBD in the presence of doxazosin at a molar ratio of 1∶5 (EphA4∶DZ). Significantly-shifted residues shared with C1 are colored in bright brown, while the residues significantly shifted only by doxazosin binding are in red. (C) The docking model of the EphA4-doxazosin complex in ribbon. Binding regions identical to those for the C1-binding were colored in brown, while those unique for the doxazosin binding in red. G, K, M and E are used to donate β-strands of the convex surface of EphA4/ephrin-binding channel. (D) EphA4 residues having direct contacts with doxazosin. Residues on D–E and J–K loops are in brown, those on the convex surface in violet, and Arg106 in cyan. Green dashed lines indicate hydrogen bonds between doxazosin and EphA4 residues. (E)–(F) The same docking model with the electrostatic potential of the EphA4 LBD displayed. (G) EphA4 LBD in free and doxazosin-bound states display different squared generalized order parameter S^2^ (Figure S6A). Blue: S^2^ difference ≤−0.01; red: S^2^ difference ≥0.01; brown: no significant change or S^2^ values not determined. (H) Conformational exchanges of EphA4 in free (left panel) and doxazosin-bound states (right panel). Residues with R_ex_>5 (Figure S6B) are displayed in balls and colored in red. (I) A docking model of the EphA2-doxazosin complex. Contact residues in D–E and J–K loops are labeled in brown, on the convex surface in cyan, and Arg103 in violet. The violet dash is used to indicate the hydrogen bonds between doxazosin and EphA2 residues.

To characterize the binding interface, we acquired a series of ^1^H-^15^N heteronuclear single quantum coherence (HSQC) spectra of the EphA4 LBD upon adding doxazosin at different molar ratios. A gradual addition of doxazosin resulted in progressive shifts of a subset of HSQC peaks (Figure 3A), consistent with the relatively low affinity interaction. Most of these HSQC peaks did not exhibit further shifts at molar ratios beyond 1∶5. Therefore, the chemical shift index (CSI) at this ratio was calculated (Figure 3B). Upon binding to doxazosin, multiple clusters of residues underwent dramatic shifts. While some of the shifts overlap with the previously described C1 antagonists of EphA4 [35], many of the shifts were unique to doxazosin (Figure 3B). The additional shifts are distributed over the convex surface of the ephrin-binding channel, including Val72-Cys73-Asn74 on E-strand, Thr104-Leu105-Arg106 on G-strand, Leu166 on K-strand and Ile192-Ala193 on M-strand. The larger contact area may account for the higher affinity of doxazosin for EphA4 than that of C1.

To visualize the EphA4-doxazosin interaction interface, we constructed models of the EphA4-doxazosin complex with the HADDOCK software in combination with CNS, as described previously for the EphA4-C1 complex [35]. The five lowest energy models are shown in Figures S5C and S5D. Doxazosin has extensive interactions with the EphA4 residues on the D–E and J–K loops, as well as contacts with the convex β-strands composed of Val72-Cys73-Asn74, Thr104-Leu105-Arg106 and Ile192-Ala193 (Figure 3C,D). Figure 3E highlights the interactions between the two doxazosin methoxy groups and EphA4 hydrophobic residues Met60 and Ile159. Interestingly, EphA4 has an additional binding pocket characterized by a positively-charged Arg106 that surrounds the electronegative oxygen atoms in benzodioxin and carbonyl groups on doxazosin (Figure 3D,E,F). Together the structural studies demonstrate direct interactions between EphA4 and doxazosin, and the KD is in similar concentration range required for cellular activation of the receptor (Figure 2).

### Binding to doxazosin stabilizes the backbone of the EphA4 LBD

Recent evidence shows that protein dynamics beyond the static structure play an important role in various biological processes including signal transmission [36], [37], [38], [39]. To gain structural insight on how doxazosin may function as an EphA2 agonist, we characterized the backbone dynamics of the EphA4 LBD in the free state vs. that in complex with doxazosin (see Methods S1). Briefly, we measured the backbone ^15^N relaxation data T1, T2 and heteronuclear NOE values, which were then analyzed by “Model-free” formulism [40], [41], [42]. The analysis generated “squared generalized order parameters”, S^2^, which reflect the backbone rigidity on the ps-ns time scale. Figure S6A demonstrates increased S^2^ values for a larger number of residues upon EphA4 binding to doxazosin compared with free EphA4. Many of the residues with significantly higher S^2^ values were mapped to the backbone of EphA4 (Figure 3G). This observation indicates that doxazosin stabilized the backbone of the EphA4 LBD on the ps-ns time scale.

Further evidence supporting the stabilization of the EphA4 LBD by doxazosin came from the chemical exchange rate, R_ex_, that reflects the conformational changes on the µs-ms time scale of individual residues. As shown in Figure S6B, many residues across the EphA4 LBD in the free state exhibited significant R_ex_ values, indicating that they undergo extensive conformational changes. By contrast, binding to doxazosin significantly reduced R_ex_ values for most residues, except for Asn74-Val75 and Thr121. The changes in R_ex_ values were then mapped back to the EphA4 structure (Figure 3H), demonstrating dramatic decreases in conformational changes in the doxazosin-bound vs. free state. In aggregate, our structural analysis and protein dynamics modeling demonstrate that doxazosin causes significant stabilization of the EphA4 LBD, which may contribute to the agonistic functions of doxazosin.

### A new model for the EphA2-doxazosin complex predicts additional agonists

Next, we modeled EphA2-doxazosin interactions incorporating the constraints from the EphA4-doxazosin structure. Sequence and structural alignments revealed residues conserved between EphA2 and EphA4 LBDs that showed significant peak shifts in EphA4 upon binding to doxazosin. These correspond to Ile58, Met59, Val69, Cys70, Asn71, Thr101, Val102, Arg103, Arg159, Leu163, Val189 and Ala190 of EphA2. Figure 3I illustrates the structure of the EphA2-doxazosin complex built with the HADDOCK software. Similar to the EphA4-doxazosin complex, the methoxy groups of doxazosin interact with the hydrophobic surface formed by Ile58, Met59 and Ala158, while Arg103 of EphA2 interacts with the carbonyl group and the oxygen atoms of the benzodioxin part of doxazosin (Figure 3I). In addition, the benzyl ring of the benzodioxin sits in a hydrophobic cavity of EphA2 mainly constituted by Cys70, Thr101 and Ala190 side chains (Figure 3I). Remarkably, in the recently determined X-ray co-crystal structure of the EphA2-ephrin-A1 complex [24], ephrin-A1 also interacts with the hydrophobic pocket and Arg103 of EphA2 (Figure 1B), suggesting that doxazosin can recapitulate two distinct modes of receptor interactions by the native ligand.

### Doxazosin triggers EphA2-dependent inhibition of ERK and Akt kinase activities

**Figure 4:**
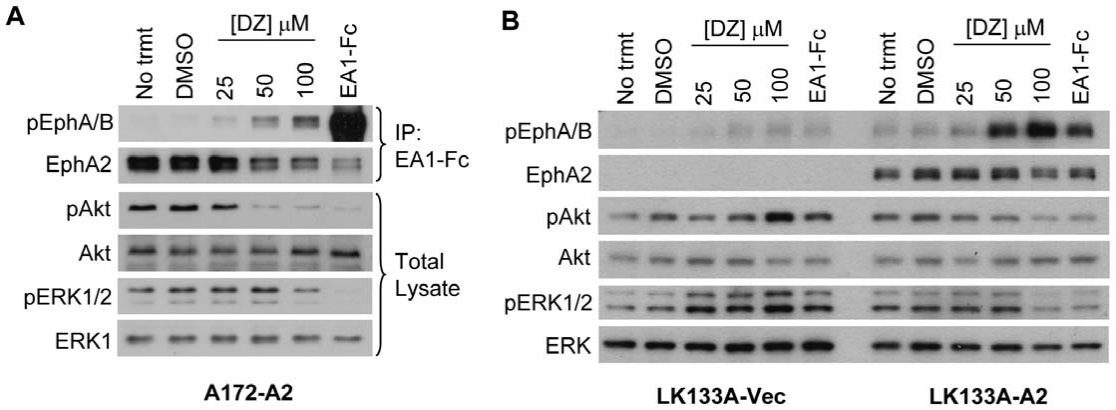
Doxazosin inhibits activation of ERK1/2 and Akt in an EphA2-dependent manner. (A) Immunoblot of lysates from A172-A2 cells treated with indicated doses of doxazosin (DZ) in 0.2% DMSO for 90 minutes. Treatment with 1 µg/ml ephrin-A1-Fc ligand for 10 minutes served as a positive control. Lysates were immunoprecipitated with ephrin-A1-Fc (IP: EA1-Fc) as given in Methods, and probed for pEphA/B and total EphA2. Total cell lysates were probed for phosphorylated forms of ERK1/2 and Akt, as well as total ERK1/2 and Akt. (B) Immunoblot of total cell lysates from LK133A-Vec and LK133A-A2 cells treated with indicated doses of doxazosin (DZ) in 0.2% DMSO for 90 minutes. Treatment with 1 µg/ml ephrin-A1-Fc for 10 minutes served as a positive control. Lysates were probed for phosphorylated forms of EphA/B, Akt, and ERK1/2, as well as total EphA2, Akt, and ERK1/2.

Activation of EphA2 receptor by ephrin-A1 ligand inhibits both ERK1/2 and Akt kinase activities in most normal cells and a subset of cancer cells [9], [12], [43]. Having demonstrated that doxazosin could mimic ephrin-A1 in binding to and activating EphA2 receptor, we asked whether doxazosin treatment could inhibit ERK1/2 and Akt activation as well. We first tested this by utilizing the A172 glioma cells engineered to overexpress EphA2 receptor (A172-A2). Unlike MDA-MB-231 cells that have activated Ras and are resistant to ephrin-A1-Fc induced inhibition of ERK1/2, A172 cells harbor wild type Ras and exhibit high basal activation levels of ERK1/2 and Akt, which were sensitive to ephrin-A1-induced inhibition (Figure 4A, far fight lane). Similar to other cell types tested (Figures 1 and 2), doxazosin also activated EphA2 on A172 glioma cells in a dose-dependent manner starting around 25 µM (Figure 4A). Moreover, treatment with 50 µM and 100 µM doxazosin was sufficient to cause significant inhibition of Akt and ERK1/2 activation. The effects on Akt inhibition coincided with EphA2 activation in these cells, and a higher concentration was needed for ERK1/2 inhibition (Figure 4A). Similar to MDA-231-A2 cells (Figure 1E), we observed degradation of EphA2 following doxazosin treatment in A172 cells.

While the results above showed that doxazosin treatment could suppress Akt and ERK activation, a key question remained whether this effect results specifically from EphA2 activation. To address this question, we utilized an immortalized liver epithelial cell line, LK133A, isolated from a hepatoma induced by DEN in EphA2 knockout mice. Retroviral vector was used to restore EphA2 expression in these cells (LK133A-A2), and cells transduced with an empty vector were used as control (LK133A-Vec). Figure 4B shows that, in the absence of EphA2, neither doxazosin nor ephrin-A1-Fc was able to inhibit ERK and Akt activities in LK133A-Vec cells (Figure 4B). In fact, there was a notable increase in ERK and Akt activation by doxazosin. Reintroduction of EphA2 expression restored ERK and Akt inhibition not only by ephrin-A1-Fc but also by doxazosin, which was accompanied by EphA2 activation. Similar to our findings in A172-A2 cells, doxazosin inhibited Akt more strongly than ERK1/2 and at a lower dose of doxazosin, again suggesting differential inhibition of these pathways by EphA2. Together, these data demonstrate that the inhibition of Akt and ERK1/2 by doxazosin is dependent on EphA2, and doxazosin is capable of triggering important downstream signaling events in a similar fashion as the native ligand, ephrin-A1.

### Doxazosin stimulates EphA2 receptor internalization and causes cell rounding similar to ephrin-A1

**Figure 5:**
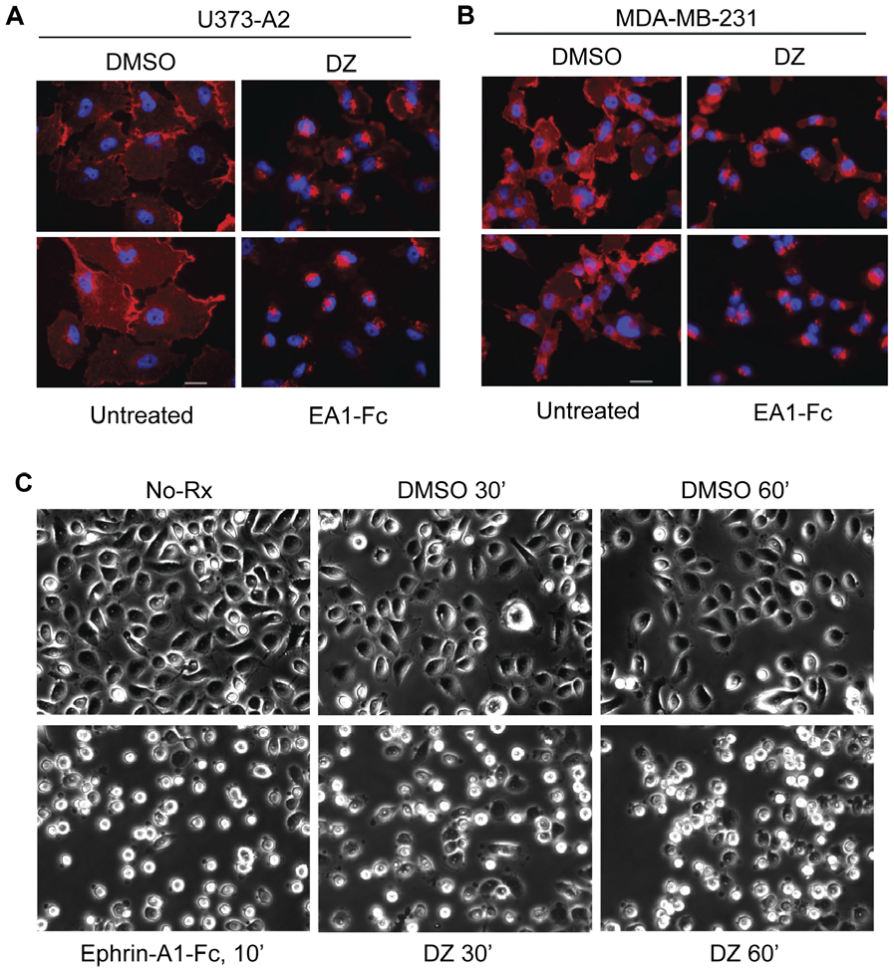
Doxazosin treatment causes EphA2 receptor internalization and induces cell rounding. (A) Immunofluorescence staining of U373-A2 cells for EphA2 receptor (red) after treatment for 60 minutes with 50 µM DZ in 0.2% DMSO. Treatment with 1 µg/ml ephrin-A1-Fc and DMSO served as positive and negative controls, respectively. DAPI nuclear staining is shown in blue. (B) Immunofluorescence staining of MDA-MB-231 cells for EphA2 receptor (red) after treatment for 120 minutes with 50 µM DZ in 0.2% DMSO. Controls are as given above. Scale bars, 25 µm. (C) Images from cell rounding analysis of PC-3 cells stimulated with 50 µM doxazosin for 30 or 60 min. Stimulation with 2 µg/ml ephrin-A1-Fc for 10 min or 0.2% DMSO served as positive and negative controls, respectively. Cells were seeded on 6-well plates and stimulated after 24 hours.

Similar to other receptor tyrosine kinases, activation of the EphA2 receptor by its ligand, ephrin-A1, results in receptor internalization and eventual degradation [44]. The decreased total level of EphA2 following doxazosin treatment in MDA-231-A2 cells (Figure 1E), A172 cells (Figure 4A), and EphA2 KO cells with restored expression (Figure 4B) suggests that the receptor is also being internalized and degraded. To directly demonstrate this at the cellular level, we used immunofluorescence to monitor localization of EphA2 following doxazosin treatment. This was first performed using the U373 glioma cell line engineered to overexpress EphA2. This cell line spreads well in culture, thereby facilitating immunofluorescence detection of cell surface and intracellular EphA2 [9]. Ephrin-A1-Fc was used as a positive control, which induced nearly complete internalization of EphA2 within 60 min (Figure 5A). Consistent with its agonistic activities, doxazosin also stimulated significant EphA2 internalization (Figure 5A). We further tested EphA2 internalization by doxazosin in parental MDA-MB-231 cells, and found that two hours of treatment with 50 µM doxazosin caused significant internalization of the endogenous EphA2, as well (Figure 5B). Together, these data confirm that doxazosin indeed triggers EphA2 receptor internalization in keeping with its agonistic activities.

As we first reported in 2000, EphA2 activation on PC-3 cells by ephrin-A1 induced rapid cell rounding, a phenomenon that is correlated with inhibition of integrin function [32]. To investigate if doxazosin could also recapitulate this aspect of native ligand function, PC-3 cells were stimulated with 50 µM doxazosin and monitored for morphological changes. We found doxazosin also caused rounding of PC-3 cells, which was similar to ephrin-A1, albeit with a slower kinetics (Figure 5C).

### Doxazosin inhibits haptotactic and chemotactic migration of multiple cancer cell types

**Figure 6:**
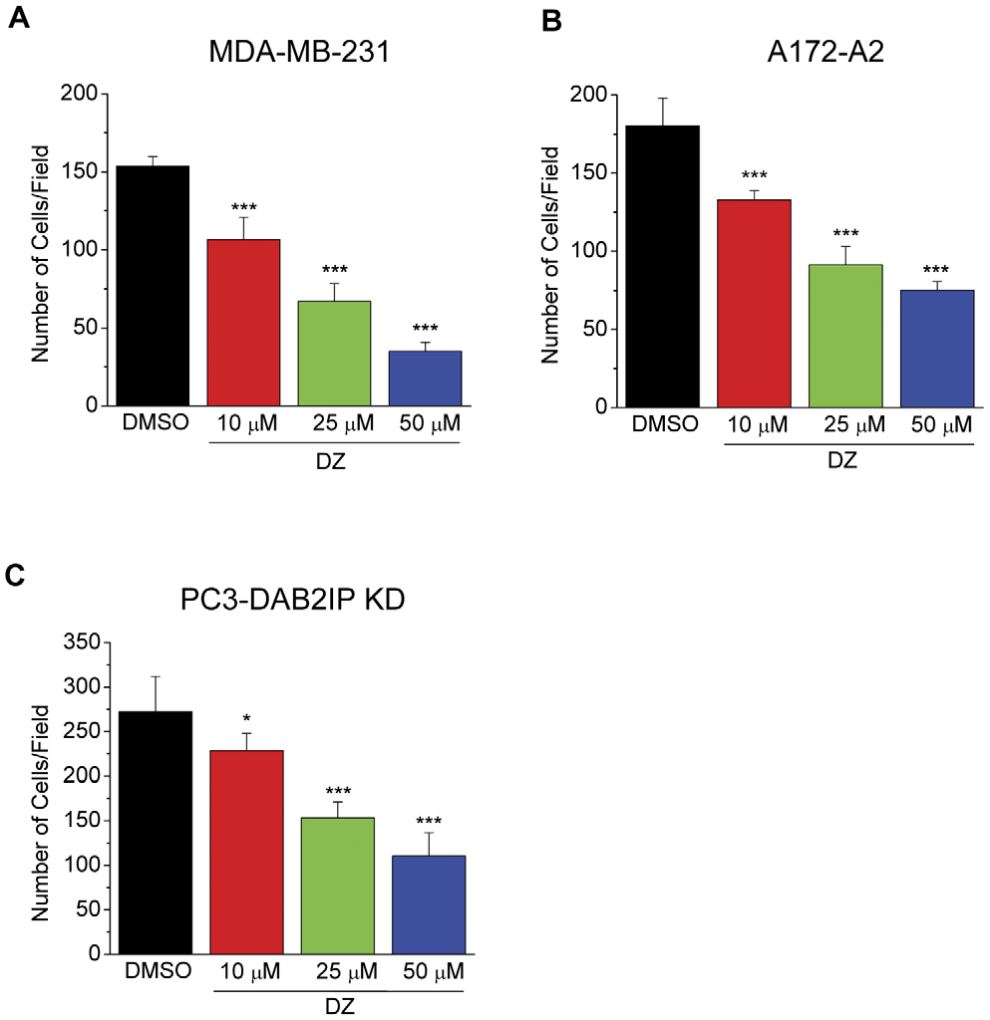
Integrin-mediated cell migration toward fibronectin is suppressed by doxazosin in a dose-dependent manner. MDA-MB-231 (A), A172-A2 (B), and PC3-DAB2IP KD (C) cells were subject to haptotactic cell migration toward fibronectin as described previously (see Methods). Doxazosin at indicated concentrations was presented at the lower chamber of the Transwells. Cells were allowed to migrate toward fibronectin for 4 hours. Data represent average numbers of migrating cells from 6 randomly selected fields. DMSO was used as vehicle control.

One of the well-established functions of Eph receptors is the ligand-dependent repulsive guidance of cell migration [17]. As a ligand-mimicking agonist for EphA2, doxazosin is expected to activate EphA2 on tumor cells and repulse migrating tumor cells *in vitro*. To test this possibility, we first investigated the effects of doxazosin on integrin-mediated haptotactic cell migration toward fibronectin in a modified Boyden chamber cell migration assay. As shown in Figure 6, a dose-dependent inhibition of cell migration was observed in MDA-MB-231 breast cancer cells (Figure 6A), A172-A2 glioma cells (Figure 6B), as well as PC-3 prostate cancer cells that were rendered highly migratory and metastatic via DAB2IP knockdown [45], [46] (PC3-DAB2IP KD, see below) (Figure 6C). The inhibitory effects were observed when doxazosin was presented either in the bottom chamber, or in both top and bottom chambers in the Transwell assay system. Next, we examined chemotactic cell migration toward hepatocyte growth factor (HGF) and found dose-dependent inhibition by doxazosin in all three cell lines as well (Figure S7), albeit to a lesser degree than those observed in the haptotactic migration assay. Therefore, doxazosin can recapitulate a key function attributed to its native ligands, i.e., negative regulation of cell migration.

### Systemic administration of doxazosin suppresses distal metastasis of prostate cancer *in vivo*

**Figure 7:**
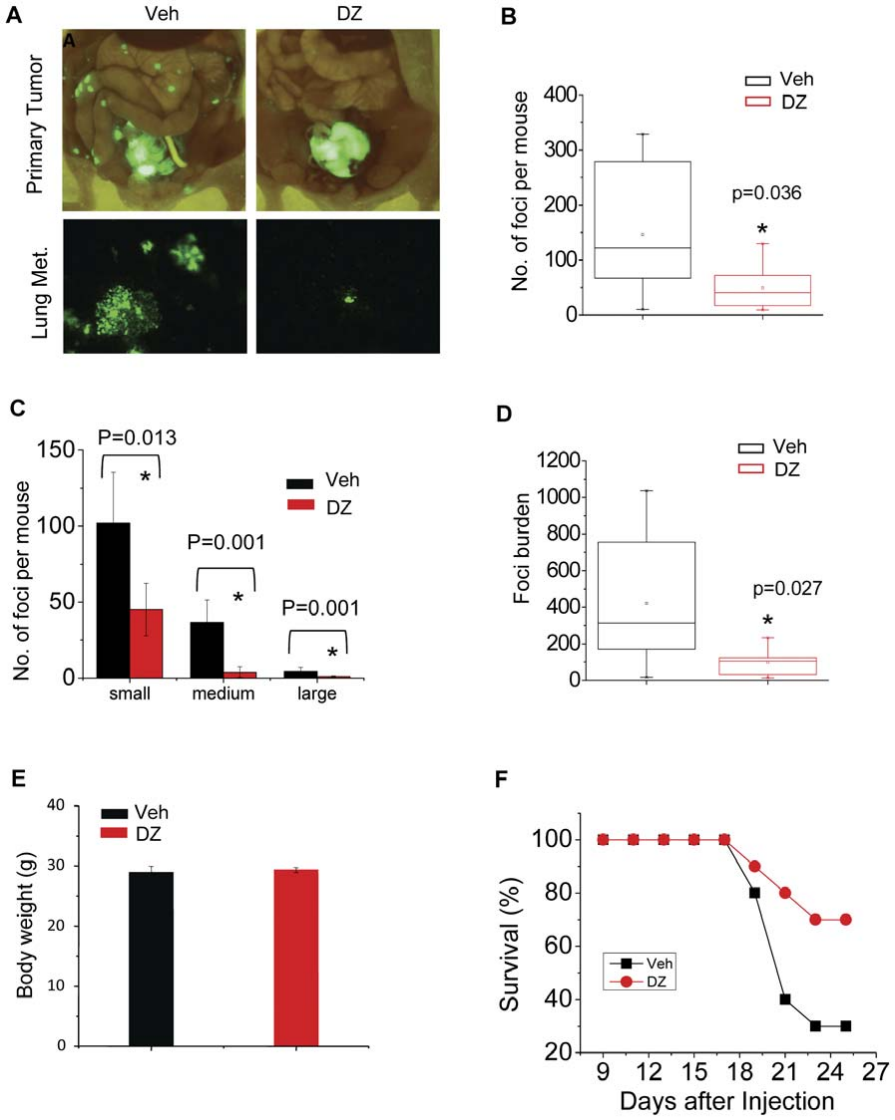
Doxazosin inhibits distal metastasis of human prostate cancer cells from orthotopic xenograft and prolongs survival. (A) Fluorescent images of prostate tumors and lung metastases resulting from GFP-tagged PC3-DAB2IP KD cells after 10 days of treatment with either vehicle, or 50 mg/kg doxazosin. Tumors in the prostate gland were imaged in a GFP light box, while lung metastatic foci were visualized under an inverted fluorescence microscope. (B) Graph comparing total number of metastatic lung foci in individual vehicle-treated (n = 7) and doxazosin-treated (n = 8) mice. (C) Quantitative analyses of total number of metastatic lung foci from different size categories. Categories were based on foci diameter measured in number of cells (small = 1–3 cells, medium = 4–6 cells, large = 7–10 cells). (D) Comparison of total metastatic burden (number of foci×foci diameter) in mice treated with vehicle control vs. those treated with doxazosin. (E) Graph comparing bodyweights of vehicle- and doxazosin-treated mice. Bars represent mean bodyweights. Error bars represent the SEM. Experiment was repeated three times with similar results. (F) Kaplan-Meier Plot showing prolonged survival in mice treated with doxazosin (n = 8) compared with those treated with vehicle control (n = 9). Mice were injected with the PC3-DAB2IP KD cells and treated as in (A) and closely monitored for survival. Those that became moribund were sacrificed. Similar results were obtained from three independent experiments.

As tumor cell migration is involved at multiple steps leading to distal tumor metastasis, the potent inhibition of cell migration by doxazosin prompted us to examine whether it could have anti-metastatic efficacy *in vivo*. Historically, studies of prostate cancer metastasis in the preclinical setting have been hampered by the lack of human cell lines that can produce significant metastasis in a reproducible manner [47]. It was recently reported that knockdown of DAB2IP, a known prostate tumor suppressor gene that is often lost in aggressive prostate cancer patients, confers PC-3 cells highly migratory and metastatic properties *in vitro* and *in vivo* [45], [46]. We took advantage of this newly established model system to test how doxazosin can affect metastasis of prostate cancer. To this end, PC3-DAB2IP KD cells were injected orthotopically into the prostate glands of nude mice. Three days later, recipient mice were subject to systemic treatment by daily i.p. injection of either vehicle control, or 50 mg/kg doxazosin for 10 days. Since these cells also expressed green fluorescent protein (GFP), freshly dissected primary tumors could be readily visualized in a GFP light box (Figure 7A top panels), and distal metastases to lungs and lymph nodes could be observed and quantified under a fluorescent microscope (Figure 7A lower panels).

Remarkably, doxazosin treatment caused a significant reduction in the number of lung metastases compared to vehicle control (Figure 7A,B). The sizes of lung metastases were also significantly smaller in doxazosin-treated mice than those from vehicle-treated mice (Figure 7C). Total metastatic burden, taking into account both numbers and sizes of metastases, was even more significantly reduced in doxazosin-treated mice (Figure 7D). Tumor cell dissemination to local lymph nodes was also reduced, but not to a significant extent. Consistent with earlier reports [33], [48], doxazosin-treated mice showed no notable side effects; there were no changes in body weight compared with vehicle control (Figure 7E), nor were there any signs of behavioral abnormalities, suggesting a lack of general toxicity at the dose used. Doxazosin has been previously shown to moderately decrease subcutaneous growth of DU145 and PC-3 cell xenografts independent of α1-adrenoreceptor [33], [49]. However, the direct target of doxazosin was not identified in those studies. A reduction in primary tumor sizes was also observed in doxazosin-treated mice, although the difference was not statistically significant (not shown), suggesting that the dramatically reduced metastasis was unlikely due to the smaller sizes of primary tumors.

Next, we determined whether doxazosin treatment might impact overall survival. Nude mice orthotopically implanted with the PC3-DAP2IP KD cells were treated for 10 days with either doxazosin, or vehicle control and monitored for survival. Consistent with earlier reports, the mice became moribund starting around three weeks after cell implantation [45], [46]. The Kaplan-Meier plot revealed an increase in overall survival of mice treated with doxazosin (70%) compared with vehicle-treated mice (30%) at 27 days after cell implantation (Figure 7F). Taken together, these preclinical data demonstrate that doxazosin can inhibit metastasis of aggressive prostate cancer from the primary site and improve overall survival in vivo.

## Discussion

Using structure-based *in silico* screening in combination with cell-based assays, we report here the identification and characterization of doxazosin as a novel EphA2 agonist that is independent of its α1-adrenoreceptor antagonist functions. Doxazosin directly bound to the recombinant EphA2 LBD with µM affinity and induced phosphorylation of EphA2 at similar doses in breast and prostate cancer cells, as well as glioma and hepatoma cells. Similar to native ligand ephrin-A1, doxazosin stimulation resulted in EphA2 internalization and degradation. Doxazosin treatment also inhibited both Akt and ERK1/2 kinase activities downstream of EphA2 activation. Consistent with the well-established ligand-dependent roles of Eph in negatively regulating cell motility, doxazosin retarded tumor cell migration *in vitro*. Moreover, in a newly established orthotopic prostate cancer metastasis model, doxazosin significantly reduced distant metastases of prostate cancer cells. We propose that, as a FDA-approved drug (Cardura®), doxazosin represents an attractive compound that can be now re-purposed for treatment of aggressive prostate cancer and potentially other malignant diseases.

Most current drug discovery efforts targeting kinases are focused on identifying small molecules that inhibit enzyme function [50]. In the case of receptor tyrosine kinases, virtually all current drug development endeavors are devoted to inhibitors targeting the ATP binding pocket. Such inhibitor-focused approaches are certainly justified in lieu of the pro-oncogenic role for most RTKs in tumors. However, the unique ligand-dependent tumor suppressor functions may make development of EphA2 agonists, rather than antagonists, a fruitful strategy for targeted therapy of a variety of solid tumors. Likewise, other kinases with intrinsic tumor suppressor functions, such as LKB1 [51] and LATS2 [52], can also be suitable targets for agonist development. The general lack of kinase agonists is often attributed to the prevailing belief that gain-of-function agonists are more difficult to develop than the loss-of-function antagonists/inhibitors. In this study, we utilized virtual screening coupled with cell-based assays to identify doxazosin as a bona fide agonist for EphA2 targeting the ligand-binding domain (LBD). Both approaches are widely utilized in contemporary drug discovery and can either be readily adapted, or developed, suggesting the feasibility to find agonists for other RTKs, including other members of the Eph subfamily.

The anti-metastatic effects of doxazosin are in keeping with the anti-migratory and anti-invasive properties of ligand activated EphA2 [3], [27]. We believe that the effects are likely to be more pronounced in tumors where the ligand-independent, pro-oncogenic functions of EphA2 predominate as a result of Akt-mediated phosphorylation of EphA2 on serine 897 [9]. The latter scenarios can take place when ligand expression is lost or reduced relative to the often overexpressed EphA2 [15], [20], [21]. Alternatively, in some cellular contexts, EphA2 can be catalytically silenced by ephrin-As on the same cells through inhibitory *cis* interactions [53], [54]. In both situations, provision of exogenous agonists acting *in trans* could still activate EphA2, unleashing its intrinsic tumor suppressor functions to inhibit tumor cell migration and invasion. It is important to note that, due to the complexity of *in vivo* settings, we cannot completely rule out the possibility that doxazosin exerts its anti-metastatic functions by affecting targets other than EphA2 or α1-adrenoreceptor.

Previous studies analyzing the crystal structure of Eph/ephrin interactions have primarily involved EphB kinases [22], [55], [56], [57]. A recent study determined the crystal structure of the ligand-binding domain of EphA2 and its interaction with ephrin-A1 [24]. The structure indicates that only minor conformational changes occur in EphA2 upon ephrin-A1 binding, suggesting that the ephrin-binding pocket on EphA2 is formed prior to ligand binding, consistent with the “lock and key” model. This is in contrast to EphB kinases, in which significant conformational changes in the ephrin-binding pocket occur following ligand binding, as a result of “induced fit” [22], [55]. This difference in binding modes contributes to the ability of ephrin-A ligands to more readily bind to their EphA receptors with much higher affinities. In addition, while the high affinity interactions with the ligand-binding channel of EphA2 are primarily involved in ephrin-A1 binding, a second low affinity binding site is involved in EphB-ephrin-B multimerization and resulting signaling [22]. In keeping with this notion, the dimeric form of ephrin-A1, and even monomeric ephrin-A1, is capable of activating EphA2 receptors, whereas multimerization of ephrin-B1 is necessary for EphB2 activation [21], [24]. Due to this enhanced binding and activation, it is suggested that EphA receptors represent better targets for small molecules than EphB receptors. Indeed, no small molecules targeting EphB receptors have yet been discovered.

Although doxazosin was discovered by virtual screening based on the EphA2 structure, it can activate EphA4 kinase at similar doses, but none of the other Eph receptors tested. This EphA2/EphA4 dual specificity has been seen previously with small molecule antagonists, which inhibited ephrin-A1 binding to both EphA2 and EphA4 kinases at high µM concentrations [58]. This shared specificity suggests structural similarities between EphA2 and EphA4 in the residues necessary for interaction with doxazosin. Given the very promiscuous nature of EphA4, which cross reacts with both ephrin-A and ephrin-B, this result is not completely surprising. While the solubility problems prevented us from directly determining the EphA2/doxazosin NMR structure, we were able to model it based on the structure of EphA4/doxazosin. The NMR structures revealed that doxazosin recapitulates both hydrophobic and electrostatic interactions of ephrin-A1 with EphA2 in the crystal structure, which could contribute to its agonistic activities.

Interestingly, both doxazosin and C1 have similar binding affinities and triggered no significant secondary structure changes of the EphA4 LBD. However, doxazosin and C1 have opposite functional effects. As revealed by the present NMR studies, in addition to contacting with the EphA4 D–E and J–K loops, doxazosin has extensive contacts with residues on the convex β-strands of the ephrin binding pocket. These interactions are observed in all complexes of Eph receptors bound to their natural ephrin ligands, but are totally lacking in the EphA4-C1 complex. This strongly suggests that designed molecules need to establish interactions with residues on the J–K, D–E loops and the convex β-strands to achieve agonistic activity. Further, the dynamic stabilization on both ps-ns and µs-ms time scales upon doxazosin binding might also play a crucial role in its agonistic activity. Although challenging, further assessment of this phenomenon may be performed by combining NMR spectroscopy and molecular dynamic (MD) simulations as we recently conducted on another system [38].

In summary, using structure-based virtual screening and cell-based assays, we have identified and characterized doxazosin as a novel small molecule agonist for EphA2. In addition, given both its ability to inhibit prostate cancer growth and its ability to inhibit prostate cancer metastasis, doxazosin may represent a cancer therapeutic agent, particularly for aggressive prostate cancer. Future optimization of the structure of doxazosin through chemical derivatization may lead to the discovery of new EphA2 agonists with enhanced affinity, specificity, and potency for use as more effective cancer therapeutics.

## Materials and Methods

### Cell lines and Reagents

Doxazosin, Labetalol, Dobutamine, and Phenoxybenzamine were all purchased from Sigma. Other compounds from the virtual screening high scoring list were obtained from Butt Park, Ltd and Chembridge Corporation. Ephrin-A1-Fc, ephrin-B1-Fc, ephrin-A5-Fc and rabbit anti-pEphA/B antibody were produced as described previously [32], [57]. Sources of antibodies include Santa Cruz Biotechnology (rabbit polyclonal anti-EphA2 and anti-ERK1, mouse anti-pERK1/2), Cell Signaling Technologies (anti-pS473-Akt and anti-Akt), R&D Systems (goat anti-EphA1, -EphA3, -EphA4, -EphB3 antibodies) and Millipore [mouse monoclonal EphA2 (clone D7)]. Rabbit polyclonal anti-EphB3 antibody was a gift from Dr. Elena Pasquale [27]. All cell lines HEK 293, MDA-MB-231, A172 and PC-3 were purchased from ATCC. PC-3-DAB2IP shRNA knockdown cells were established and described [45], [46]. Cells were maintained in either Dulbecco's Modified Eagle Medium (DMEM), or RPMI 1640 (PC-3 and PC3-DAB2IP KD) supplemented with 10% FBS, 10 mg/ml glutamine, 100 U/ml penicillin, and 0.1 mg/ml streptomycin.

### *In silico* screening of compounds

For *in silico* screening we have used compounds from the NCI database (∼250000 compounds), Sigma database of rare chemicals (∼100000 compounds) and Available Chemical Directory (ACD) (http://mdl.com) (∼350,000 commercially available). A total of ∼700,000 molecules were preprocessed before docking using the FILTER utility from the OpenEye software package (http://eyesopen.com) in order to eliminate toxic or reactive chemicals and molecules without drug-like properties. This left about 100,000 compounds for which multiple conformations were generated using the OMEGA utility (OpenEye) for rigid docking. The Gasteiger-Marsili atomic charges were assigned using BABEL freeware utility. The 3D structure of the EphA2 receptor ligand binding domain was obtained by homology modeling as described previously [23].

The *in silico* screening was done using DOCK5.1 software. DOCK5.1 implements a geometry-based approach for the docking of the small ligands [59]. Connolly molecular surface [60] of the EphA2 receptor structure was calculated using a probe radius of 1.4 Å. The negative image of the binding site was created using the SPHGEN module, which generates the spheres of a certain radii and calculates the best match of these spheres with the molecular surface of the protein docking site. The DOCK module was used to fit the small molecules from the database to the centers of these spheres and the best obtained configurations were scored in terms of energy using the AMBER force field [61]. The virtual screening was carried out on Beowulf cluster of Linux machines at the Ohio Supercomputer Center.

### Surface Plasmon Resonance Analysis

Binding analysis was carried out using standard biosensor chips and the SR7000DC Dual Channel SPR System (Reichert Life Sciences, Depew, NY). The extracellular domain (ECD) of EphA2 was coupled to the chip by standard amine coupling using 1-ethyl-3-(3-dimethylaminopropyl) carbodiimide (EDC) and N-hydroxysuccinimide (NHS), followed by ethanolamine wash. In the case of doxazosin, Neutravidin was coupled to the chip, followed by ethanolamine wash and addition of minimally biotinylated EphA2 ECD to properly orient the protein. Different concentrations of dobutamine, labetalol, and doxazosin in running buffer (PBS, 0.05% Tween-20, 1% DMSO) were flown over the chip at a rate of 75–90 µl/min and binding analyzed in real-time. After approximately 60 seconds, running buffer was flown over the chip to dissociate the compounds from the coupled protein (downward slope of curve). Binding data were analyzed and K_D_ values determined using Clamp software (BioLogic Software Pty Ltd).

### Cell stimulation, immunoprecipitation, immunoblotting, and immunofluorescence staining

Cells were plated in 24-well dishes at a density of 100,000 cells/well and grown for 24 hours prior to stimulation with appropriate compounds and ephrins for the given amounts of time. Compounds were prepared in DMSO at 500 times the final concentrations and 0.2% DMSO was used as vehicle control. Following treatment, cells were lysed directly in SDS Gel Loading Buffer and immunoblotted as described previously [32]. Immunoprecipitation and immunofluorescence staining was performed as described [32]. For immunoprecipitation, 300,000 cells/well were plated in 6-well dishes and lysed in modified RIPA Buffer (20 mM Tris-HCl pH 7.4, 20 mM NaF, 150 mM NaCl, 10% glycerol, 0.1% SDS, 0.5% DCA, 2 mM EDTA, 1% Triton X-100) following treatment. Either 10 µg of ephrin-A1-Fc, or 4 µg of EphA2 mouse monoclonal antibody (Clone D7, Millipore) were used, and mouse IgG was used as a negative control for EphA2 IP.

### ITC and NMR characterization of the binding of the EphA4 LBD with doxazosin

Production of the 181-residue EphA4 ligand binding domain (LBD, residues 29–209) and isothermal titration calorimetry (ITC) experiments were performed as previously described [35]. To characterize the binding of the EphA4 LBD and doxazosin by NMR, two-dimensional ^1^H-^15^N HSQC spectra of the ^15^N-labeled EphA4 LBD were acquired at 25°C with a protein concentration of 100 µM in the absence and presence of Doxazosin at molar ratios of 1∶1; 1∶2, 1∶4, 1∶5 and 1∶6 (EphA4/DZ). By superimposing HSQC spectra, the shifted HSQC peaks could be identified and further assigned to the corresponding residues of the EphA4 ectodomain. The degree of perturbation was measured by an integrated chemical shift index (CSI) calculated by the formula [(ΔH)^2^+(ΔN)^2^/5]^1/2^ [35].

### *In vivo* metastasis assay

PC-3 cells (from ATCC) engineered to express both GFP and DAB2IP shRNA were characterized previously [46]. Cells were suspended in serum free medium and 2,000 cells injected directly into the prostate glands of 7 week old NCr athymic *nu/nu* mice. Three days after injection, mice were divided into two groups, one receiving vehicle alone (5% DMSO/10% Cremophor/PBS) and another 50 mg/kg doxazosin via intraperitoneal injection daily for 10 days. Mice were sacrificed 14 days later and intact primary tumors were imaged under a UV light box with a Digital camera. Lungs were dissected into separate lobes and GFP-expressing metastases were imaged using an inverted fluorescent microscope (Leica). Metastases were enumerated in the two largest lung lobes and placed into size categories based upon their diameters, measured in number of cells (1–10 cells). Total metastatic burden was calculated in each mouse using the following equation: [burden = ∑(# of foci×focus diameter)].

All procedures involving mice were performed in accordance with guidelines set forth by the American Association for Accreditation of Laboratory Animal Care and the USPHS “Policy on Humane Care and Use of Laboratory Animals”. Studies were approved and supervised by The Case Western Reserve University Institutional Animal Care and Use Committee.

### Statistical Analyses

Significance of results was determined using Student's two-tailed t-test. Differences were considered significant when P≤0.05.

## Supporting Information

Figure S1 MDA-231-EphA2 cells overexpress EphA2. Representative immunoblot for activated Eph receptors (pEphA/B) and total EphA2 on MDA-231-Vector and MDA-231-EphA2 cells treated for given times with 1 µg/ml ephrin-A1-Fc ligand (EA1-Fc). Blotting for total ERK served as a loading control.
(TIF)

Figure S2 Chemical structures of compounds screened for Eph kinase activation.
(TIF)

Figure S3 EphA2 binding and receptor activation by dobutamine and labetalol. (A) Immunoblots for pEphA/B on lysates from MDA-231-A2 cells treated for 30 minutes with indicated doses of dobutamine (DB) and labetalol (LB) in 0.2% DMSO. Blotting for total EphA2 served as a loading control. Structures of DB and LB are shown below respective blots. (B) Representative plots from Surface Plasmon Resonance (SPR) analysis of DB and LB binding to the recombinant extracellular domain (ECD) of EphA2 kinase. Curves from bottom to top represent concentrations of 62.5, 125, 250, 500, 1000 µM. Determined K_D_ values are shown within each plot.
(TIF)

Figure S4 Comparison of the EphA2 ligand-binding domain (LBD) homology model and crystal structure. Ribbon diagrams of both the homology model (red) and crystal structure (yellow) of the EphA2 LBD. Overlay of the homology model and crystal structure is shown on the far right.
(TIF)

Figure S5 Biophysical characterization of the EphA4 and doxazosin interaction. (A) ITC profile of the binding reaction of the EphA4 LBD with doxazosin (top) and integrated values for reaction heats with subtraction of the corresponding blank results normalized by the amount of ligand injected vs the molar ratio of EphA4/DZ (bottom). The thermodynamic binding parameters obtained from fitting the data are shown in the box. (B) Far-UV circular dichroism spectra of the EphA4 LBD in the absence (blue) and in the presence of doxazosin (red) at a molar ratio of 1∶5 (EphA4∶DZ). (C)–(D) Superimposition of five lowest-energy docking structures of the EphA4-doxazosin complex.
(TIF)

Figure S6 ^15^N backbone dynamics of the free and doxazosin-complexed EphA4 LBD. (A) Differences of the squared generalized order parameters (S^2^) between the EphA4-doxazosin complex and free EphA4 LBD. The bars with positive values are colored in red while ones with negative values in blue. (B) R_ex_ values derived from the model-free analysis of the relaxation data. Data for the free EphA4 LBD are colored in blue while those for the EphA4 in complex with doxazosin are colored in red.
(TIF)

Figure S7 Inhibition of chemotactic cell migration of PC3-DAB2IP KD, A172-A2, and SCP3-231 cells upon doxazosin treatment. PC3-DAB2IP KD, A172-A2, and SCP3-231 cells were subject to chemotactic cell migration toward 15 ng/ml hepatocyte growth factor (HGF) as described previously (see Methods S1). HGF and doxazosin at indicated concentrations were presented in the lower chamber of the Transwells. Cells were allowed to migrate toward HGF for 5 hours. Data represent average numbers of migrating cells from 6 randomly selected fields. DMSO was used as vehicle control.
(TIF)

Methods S1 NMR experiments for relaxation data acquirement.
(DOC)
