# Supplementary figures and images for: A Small Molecule Agonist of EphA2 Receptor Tyrosine Kinase Inhibits Tumor Cell Migration In Vitro and Prostate Cancer Metastasis In Vivo
Source: PLoS One. 2012 Aug 15;7(8):e42120. doi: 10.1371/journal.pone.0042120 (PMC3419725; doi:10.1371/journal.pone.0042120)

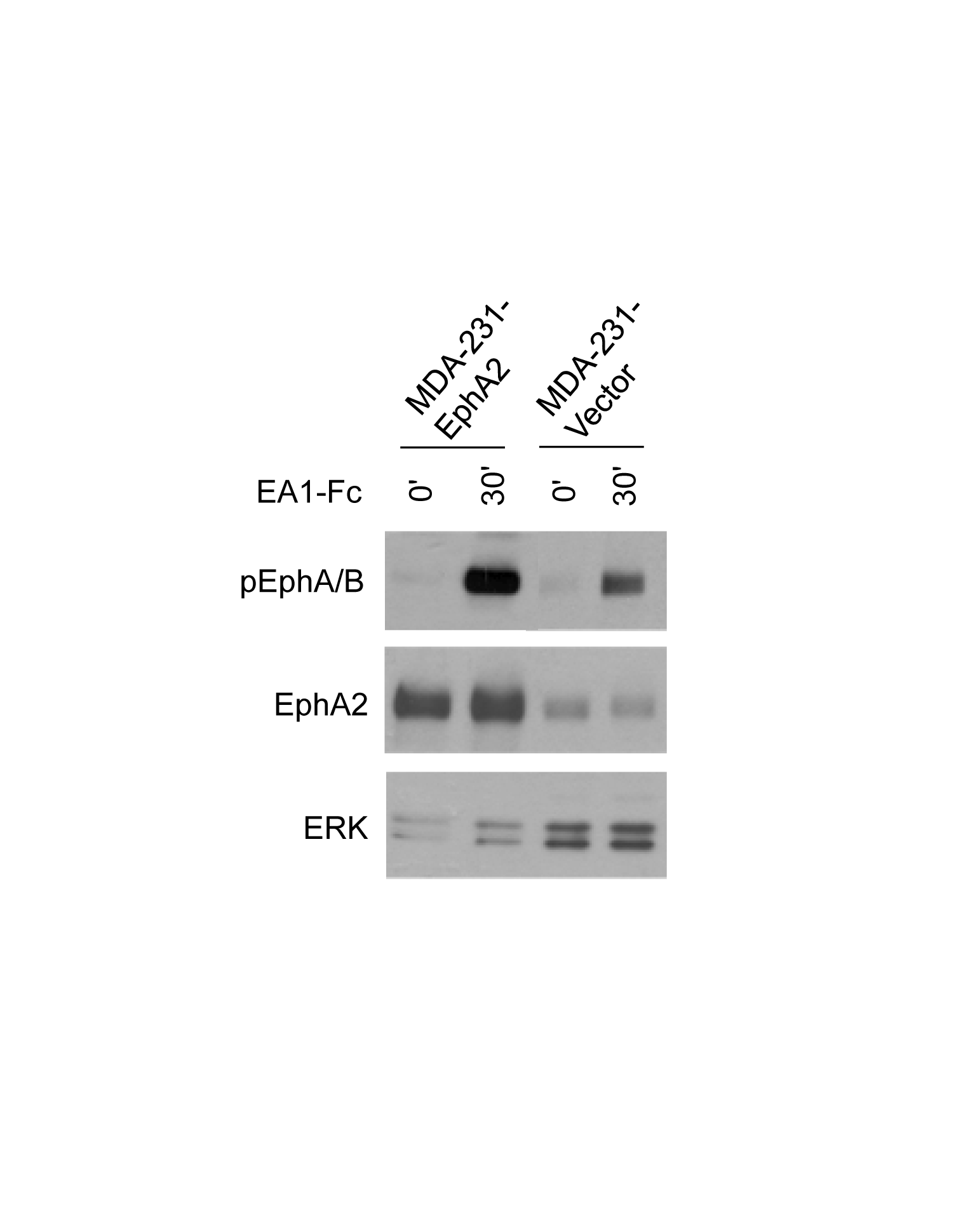

Supplement: Figure S1 — MDA-231-EphA2 cells overexpress EphA2. Representative immunoblot for activated Eph receptors (pEphA/B) and total EphA2 on MDA-231-Vector and MDA-231-EphA2 cells treated for given times with 1 µg/ml ephrin-A1-Fc ligand (EA1-Fc). Blotting for total ERK served as a loading control. (TIF) [file pone.0042120.s001.tif]

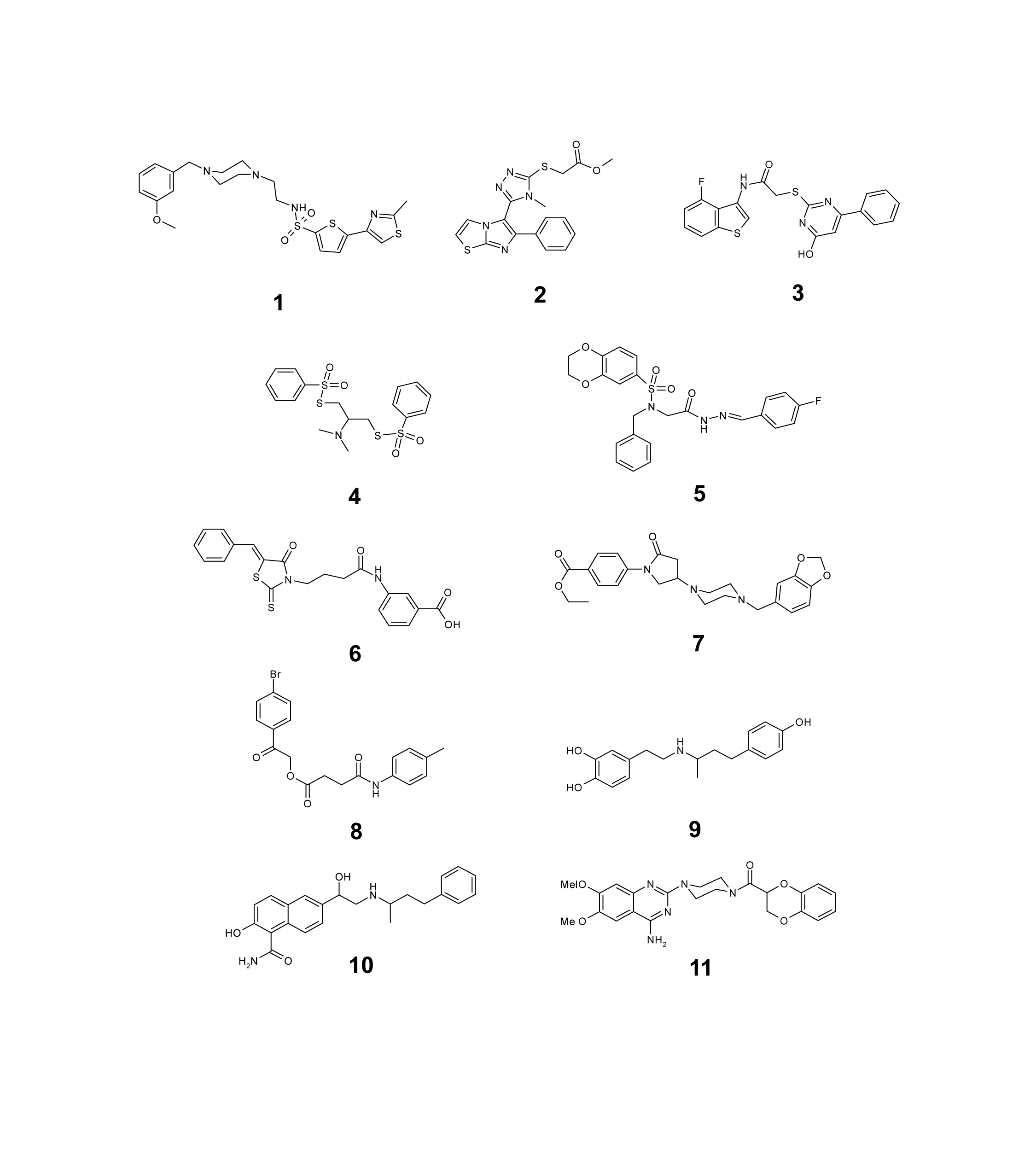

Supplement: Figure S2 — Chemical structures of compounds screened for Eph kinase activation. (TIF) [file pone.0042120.s002.tif]

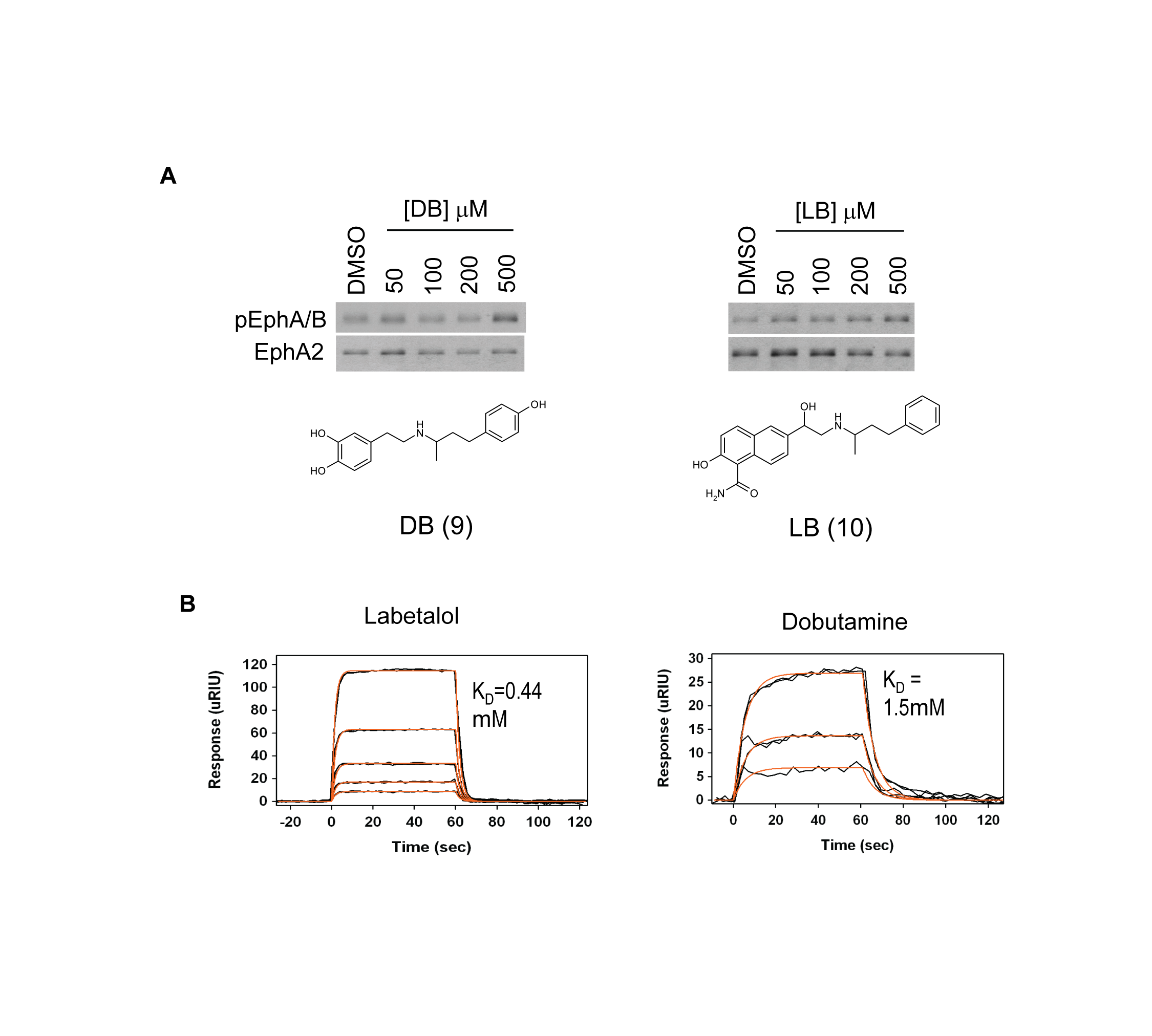

Supplement: Figure S3 — EphA2 binding and receptor activation by dobutamine and labetalol. (A) Immunoblots for pEphA/B on lysates from MDA-231-A2 cells treated for 30 minutes with indicated doses of dobutamine (DB) and labetalol (LB) in 0.2% DMSO. Blotting for total EphA2 served as a loading control. Structures of DB and LB are shown below respective blots. (B) Representative plots from Surface Plasmon Resonance (SPR) analysis of DB and LB binding to the recombinant extracellular domain (ECD) of EphA2 kinase. Curves from bottom to top represent concentrations of 62.5, 125, 250, 500, 1000 µM. Determined KD values are shown within each plot. (TIF) [file pone.0042120.s003.tif]

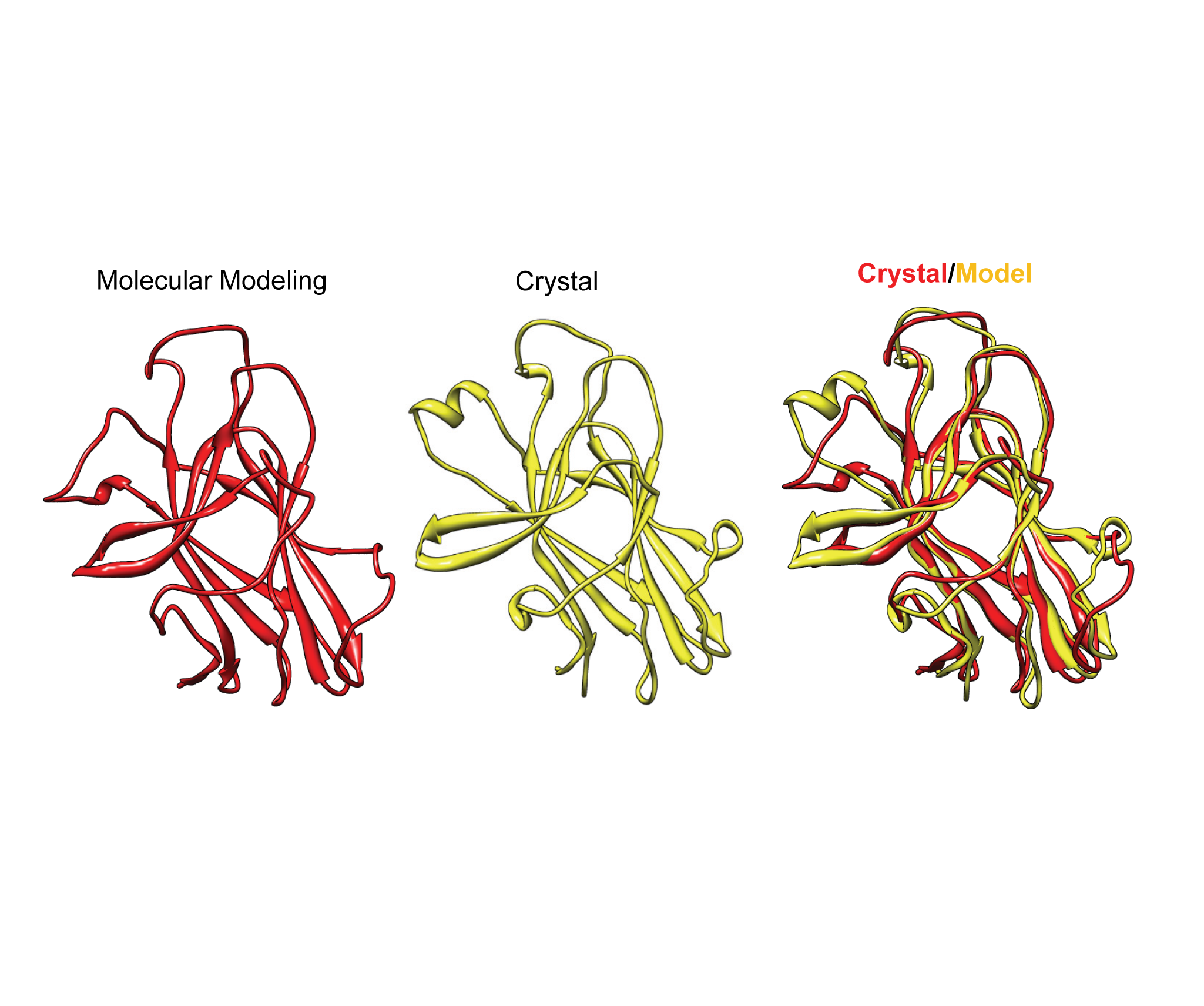

Supplement: Figure S4 — Comparison of the EphA2 ligand-binding domain (LBD) homology model and crystal structure. Ribbon diagrams of both the homology model (red) and crystal structure (yellow) of the EphA2 LBD. Overlay of the homology model and crystal structure is shown on the far right. (TIF) [file pone.0042120.s004.tif]

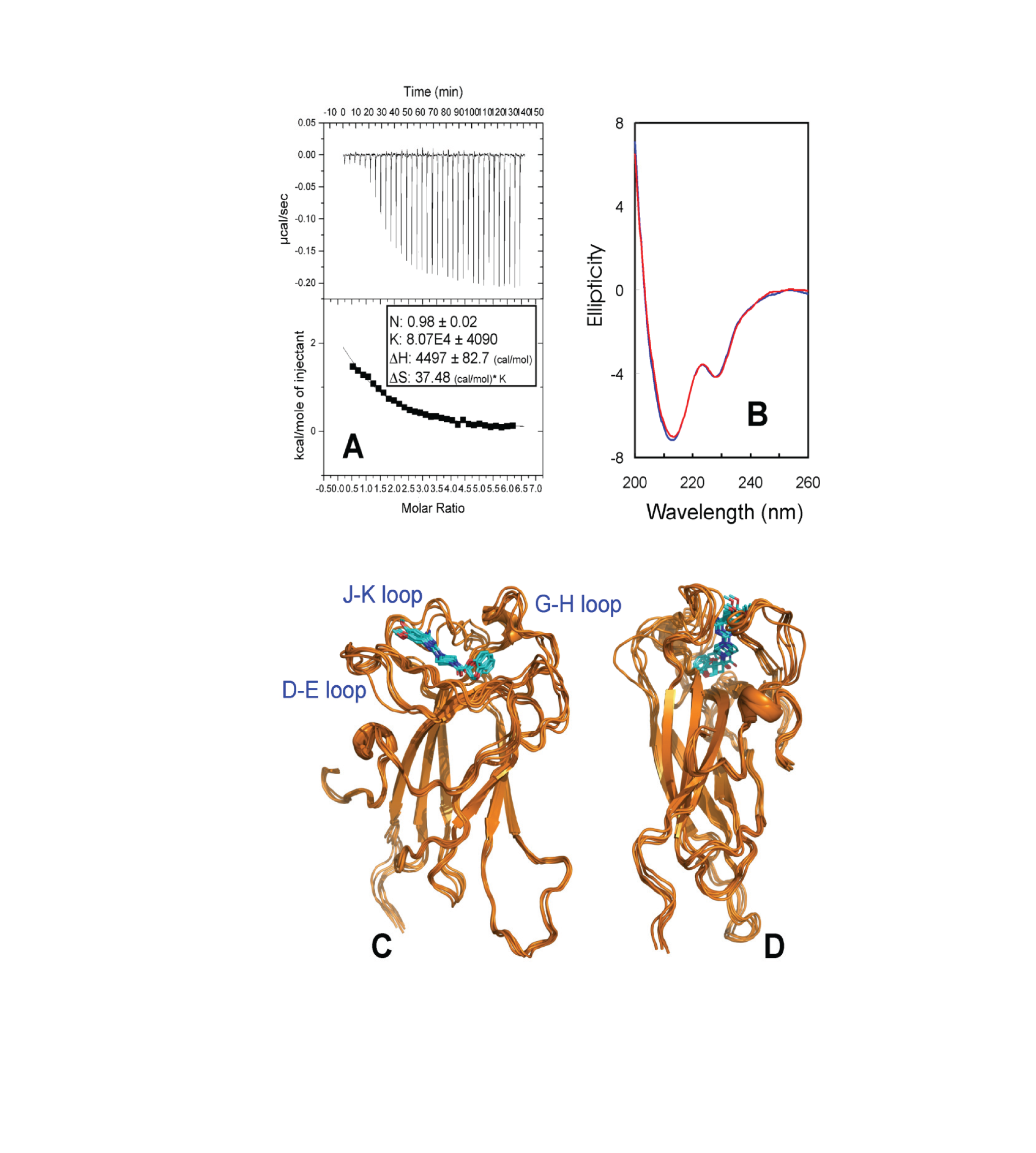

Supplement: Figure S5 — Biophysical characterization of the EphA4 and doxazosin interaction. (A) ITC profile of the binding reaction of the EphA4 LBD with doxazosin (top) and integrated values for reaction heats with subtraction of the corresponding blank results normalized by the amount of ligand injected vs the molar ratio of EphA4/DZ (bottom). The thermodynamic binding parameters obtained from fitting the data are shown in the box. (B) Far-UV circular dichroism spectra of the EphA4 LBD in the absence (blue) and in the presence of doxazosin (red) at a molar ratio of 1∶5 (EphA4∶DZ). (C)–(D) Superimposition of five lowest-energy docking structures of the EphA4-doxazosin complex. (TIF) [file pone.0042120.s005.tif]

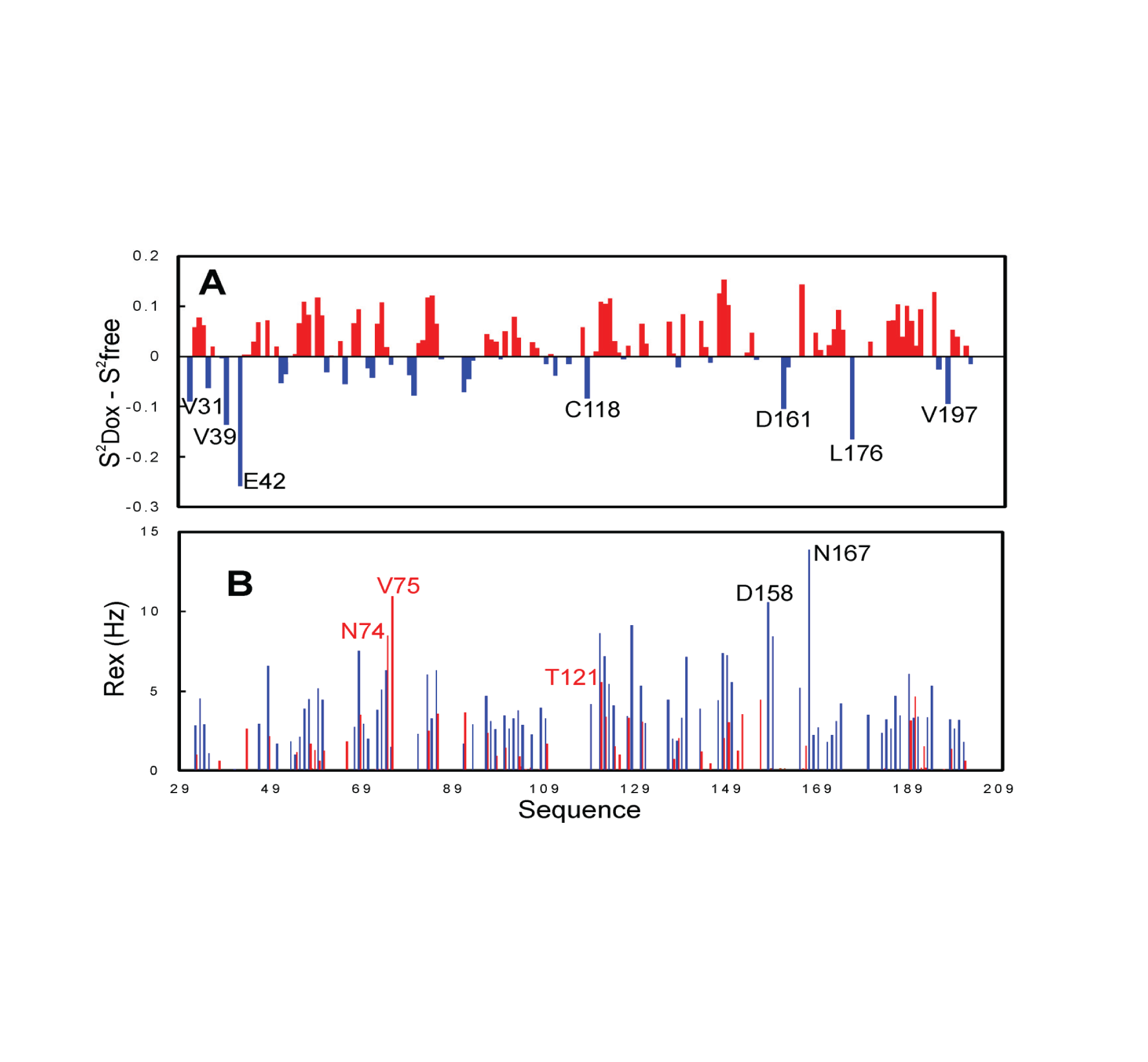

Supplement: Figure S6 — 15N backbone dynamics of the free and doxazosin-complexed EphA4 LBD. (A) Differences of the squared generalized order parameters (S2) between the EphA4-doxazosin complex and free EphA4 LBD. The bars with positive values are colored in red while ones with negative values in blue. (B) Rex values derived from the model-free analysis of the relaxation data. Data for the free EphA4 LBD are colored in blue while those for the EphA4 in complex with doxazosin are colored in red. (TIF) [file pone.0042120.s006.tif]

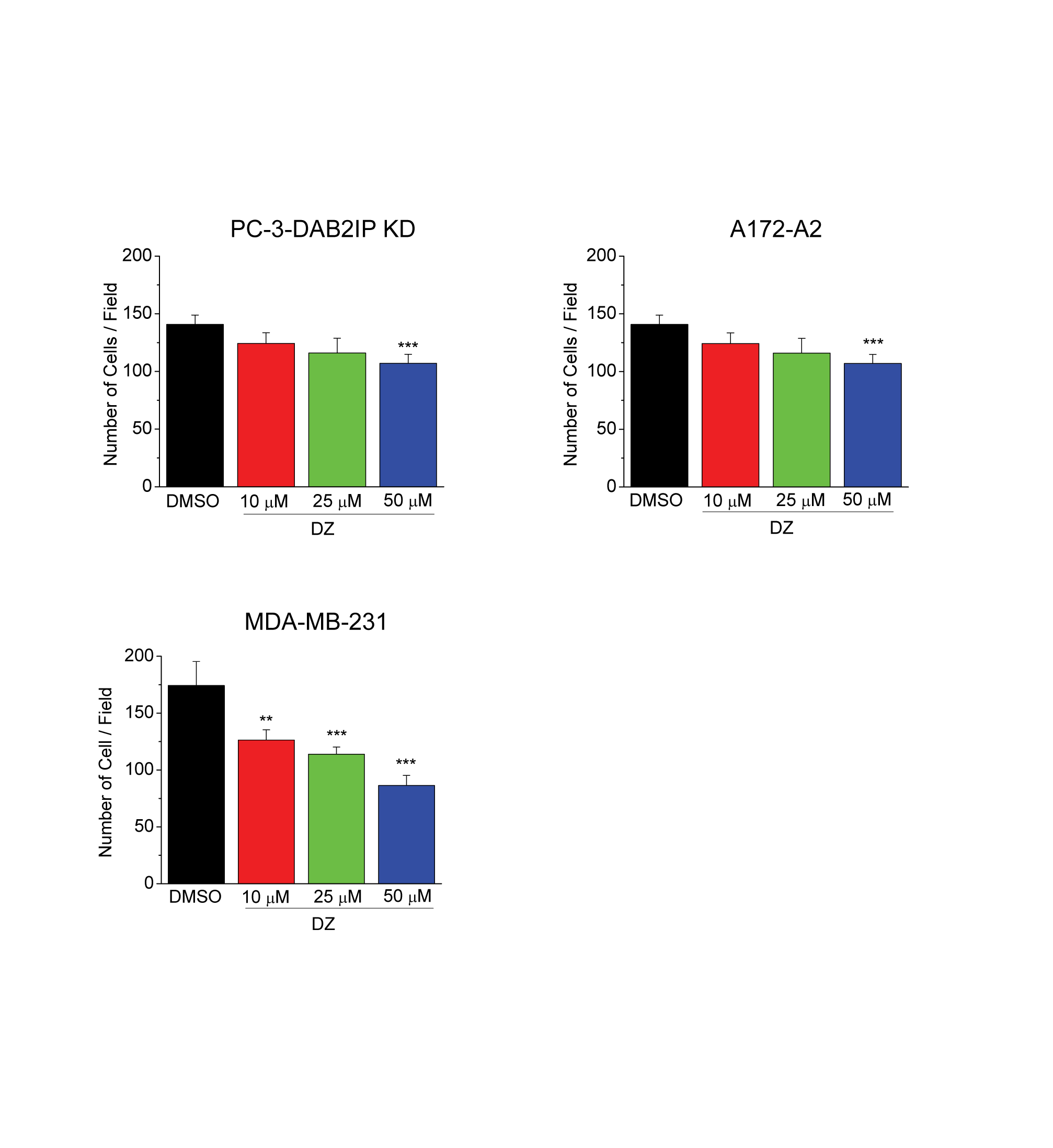

Supplement: Figure S7 — Inhibition of chemotactic cell migration of PC3-DAB2IP KD, A172-A2, and SCP3-231 cells upon doxazosin treatment. PC3-DAB2IP KD, A172-A2, and SCP3-231 cells were subject to chemotactic cell migration toward 15 ng/ml hepatocyte growth factor (HGF) as described previously (see Methods S1). HGF and doxazosin at indicated concentrations were presented in the lower chamber of the Transwells. Cells were allowed to migrate toward HGF for 5 hours. Data represent average numbers of migrating cells from 6 randomly selected fields. DMSO was used as vehicle control. (TIF) [file pone.0042120.s007.tif]
