# Supplementary material for: A Small Molecule Agonist of EphA2 Receptor Tyrosine Kinase Inhibits Tumor Cell Migration In Vitro and Prostate Cancer Metastasis In Vivo
Source: PLoS One. 2012 Aug 15;7(8):e42120. doi: 10.1371/journal.pone.0042120 (PMC3419725; doi:10.1371/journal.pone.0042120)
Supplement: Methods S1 — NMR experiments for relaxation data acquirement. (DOC) [file pone.0042120.s008.doc]

**SUPPORTING INFORMATION**

**A Small Molecule Agonist of EphA2 Receptor Tyrosine Kinase Inhibits Tumor Cell Migration in vitro and Prostate Cancer Metastasis in vivo**

Aaron Petty ^1*^, Eugene Myshkin ^1*^, Haina Qin ^2*^, Hong Guo ^1^, Hui Miao ^1^, Gregory P. Tochtrop ^3,4^ Jer-Tsong Hsieh ^5^, Phillip Page ^6^, Lili Liu ^7^, Daniel J. Lindner ^8^, Chayan Acharya ^9^, Alexander D. MacKerell, Jr. ^9^, Eckhard Ficker ^1^, Jianxing Song ^2,10#^, and Bingcheng Wang^1,4,11,#^

***** These authors contributed equally to this work

^1^ Rammelkamp Center for Research and Department of Medicine, MetroHealth Campus; Case Western Reserve University School of Medicine, Cleveland, OH , United States of America

^2^ Departments of Biological Sciences, Faculty of Science, National University of Singapore, Singapore

^3^ Department of Chemistry, Case Western Reserve University, Cleveland, OH, United States of America

^4^ Case Comprehensive Cancer Center, Case Western Reserve University School of Medicine, Cleveland, OH, United States of America

^5^ Department of Urology, University of Texas Southwestern Medical Center, Dallas, TX, United States of America

^6^ Reichert, Inc., Depew, NY, United States of America

^7^ Department of Medicine, Division of Hematology and Oncology, University Hospitals Case Medical Center, Case Western Reserve University School of Medicine, Cleveland, OH, United States of America

^8^ Taussig Cancer Institute, Cleveland Clinic Foundation, Cleveland, OH, United States of America

^9^ Department of Pharmaceutical Sciences, School of Pharmacy, University of Maryland, Baltimore, MD, United States of America

^10^ Department of Biochemistry, Yong Loo Lin School of Medicine, National University of Singapore, 10 Kent Ridge Crescent, Singapore

^11^ Department of Pharmacology, Case Western Reserve University School of Medicine, Cleveland, OH, United States of America

Keywords: EphA2, agonist, kinase, metastasis, small molecules

^+^ Corresponding author. Tel: +1-216-778-4256; Fax: +1-216-778-4321; E-mail: [bxw14@case.edu](mailto:bxw14@case.edu)

^+^ Corresponding author. Tel: +65-6874-1013; Fax: +65-67792486; E-mail: [bchsj@nus.edu.sg](mailto:bchsj@nus.edu.sg)

**SUPPORTING METHODS**

**NMR experiments for relaxation data acquirement**

^15^N T1, T1ρ relaxation times, {^1^H}-^15^N steady state NOE intensities were collected on an 800 MHz Bruker Avance spectrometer equipped with both an actively shielded cryoprobe and pulse field gradient units [1], [2]. Relaxation times T1 were determined by collecting 8 points with delays of 10, 280, 700, 1000, 1100, 1250, and 1400 ms using a recycle delay of 1 s. Relaxation times T1ρ were measured by collecting seven points with delays of 1, 10, 20, 30, 40, 45, and 52 ms using a spin-lock power of 1.6 kHz, a 2.5-s recycle delay. {^1^H}-^15^N steady-state NOEs were obtained by recording spectra with and without ^1^H presaturation of a duration of 3 s plus a relaxation delay of 6 s at 800 MHz.

Relaxation times were fitted as single exponential decays to peak height data. Spin-spin relaxation time T2 was calculated from T1ρ and T1 according to equation:


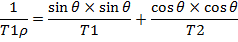


Where θ=atan(Δω/ω_1_) and Δω, ω_1_ are the resonance offset and spin-lock field strength, respectively [1] . Due to the overlap or/and weak intensity of many resonance peaks resulting from the relatively-large size and presence of many exposed loop residues, 127 and 122 out of 175 non-Proline peaks are appreciable for the quantitative analysis in the free state and in complex with doxazosin, respectively.

**Model-free analysis**

NMR relaxation data were analyzed by “Model-Free” formulism with protein dynamics software suites [3]. Briefly, relaxation of protonated heteronuclei is dominated by the dipolar interaction with the directly attached ^1^H spin and by the chemical shift anisotropy mechanism [4]. Relaxation parameters are given by:


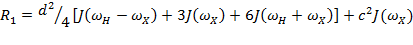


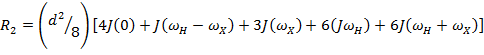


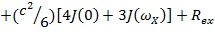


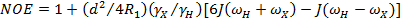


In which
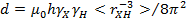
,
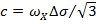
,
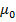
 is the permeability of free space; *h* is Planck’s constant;
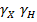
 are the gyromagnetic ratios of ^1^H and the X spin (X=^13^C or ^15^N) respectively;
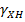
 is the X-H bond length;
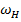
 and
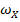
 are the Larmor frequencies of ^1^H and X spins, respectively; and Δσ is the chemical shift anisotropy of the X spin.

The Model-Free formalism, as previously established [5] and further extended [6], determines the amplitudes and time scales of the intramolecular motions by modeling the spectral density function, *J*(*ω*), as


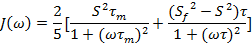


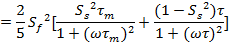


In which,
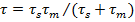
,
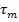
 is the isotropic rotational correlation time of the molecule,
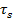
 is the effective correlation time for internal motions,
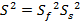
 is the square of the generalized order parameter characterizing the amplitude of the internal motions, and
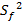
 and
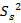
 are the squares of the order parameters for the internal motions on the fast and slow time scales, respectively. Generalized order parameters reflect motions on the ns-ps time scale, with values ranging from zero for isotropic internal motions to unity for completely restricted motion in a molecular reference frame.

In order to allow for diverse protein dynamics, several forms of the spectral density function, based on various models of the local motion [7], were utilized, which include the original Lipari-Szabo approach, assuming fast local motion characterized by the parameters *S*^2^ and *τ_loc_*; extended model-free treatment, including both fast (
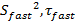
) and slow (
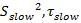
) reorientations for the NH bond (
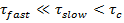
); and could also allow for slow, milli- to microsecond dynamics resulting in a conformational exchange contribution, *R_ex_*, to the linewidth. Here, analysis of relaxation data was performed by software DYNAMICS [3], [8].

The overall rotational diffusion tensors as well as total correlation time (τc) of the EphA4 LBD in the free state and complex with doxazosin were determined by ROTDIF [3], [9]. Isotropic, axially-symmetric and fully anisotropic models for the overall motion were used and then compared. According to the illustration of ROTDIF, fully anisotropic model was finally selected because of smallest Ch^2^/df value.

**Chemotactic Cell Migration Assay**

Chemotactic cell migration assay was performed as described previously [10]. Briefly, both sides of a Transwell insert were coated with 10 µg/ml rat tail Collagen I overnight at 4^o^ C. Seventy thousands of cells were placed into the upper chamber and serum-free medium containing both 15 ng/ml hepatocyte growth factor (HGF) and doxazosin at indicated concentrations were added to the lower chamber. Cells were allowed to migrate for 5 hours. At the end of migration, cells were fixed with 4% paraformaldehyde and stained with 0.5% crystal violet. Cells that have migrated to the bottom of the Transwell insert were counted from 6 random fields.

**SUPPORTING REFERENCES**

1. Fan D, Zheng Y, Yang D and Wang J (2003) **NMR solution structure and dynamics of an exchangeable apolipoprotein, Locusta migratoria apolipophorin III.** J Biol Chem 278: 21212–21220.
2. Ran X, Qin H, Liu J, Fan JS, Shi J, et al. (2008) NMR structure and dynamics of human ephrin-B2 ectodomain: The functionally critical C-D and G-H loops are highly dynamic in solution. Proteins 72: 1019–1029.
3. Fushman D, Cahill S, Cowburn D (1997) The main-chain dynamics of the dynamin pleckstrin homology (PH) domain in solution: analysis of 15N relaxation with monomer/dimer equilibration. J Mol Biol 266: 173-194.
4. Abragam A (1961) Principles of Nuclear Magnetism. Oxford, Clarendon Press.
5. Lipari G & Szabo A (1982) Model-free approach to the interpretation of Nuclear Magnetic Resonance relaxation in macromolecules. 1. Theory and range of validity. J Am Chem Soc 104: 4546-4559.
6. Clore GM, Szabo A, Bax A, Kay LE, Driscoll PC, et al. (1990) Deviations from the simple two-parameter model-free approach to the interpretation of Nitrogen-15 Nuclear Magnetic Relaxation of proteins. J Am Chem Soc 112: 4989-4991.
7. Palmer AG, Rance M & Wright PE (1991) Intramolecular motions of a zinc finger DNA-binding domain from Xfin characterized by Proton-Detected Natural Abundance 3C Heteronuclear NMR Spectroscopy. J Am Chem Soc 113: 4371-4380.
8. Hall JB, Fushman D (2003) Characterization of the overall and local dynamics of a protein with intermediate rotational anisotropy: Differentiating between conformational exchange and anisotropic diffusion in the B3 domain of protein G. J Biomol NMR 27: 261-275
9. Walker O, Varadan R, Fushman D (2004) Efficient and accurate determination of the overall rotational diffusion tensor of a molecule from (15)N relaxation data using computer program ROTDIF. J Magn Reson 168: 336-345.
10. Miao H, Nickel CH, Cantley LG, Bruggeman LA, Bennardo LN, et al. (2003) EphA kinase activation regulates HGF-induced epithelial branching morphogenesis. J Cell Biol 162: 1281-1292.
